# The Annual American Men's Internet Survey of Behaviors of Men Who Have Sex With Men in the United States: Protocol and Key Indicators Report 2013

**DOI:** 10.2196/publichealth.4314

**Published:** 2015-04-17

**Authors:** Travis Howard Sanchez, R Craig Sineath, Erin M Kahle, Stephen James Tregear, Patrick Sean Sullivan

**Affiliations:** ^1^ Emory University Atlanta, GA United States; ^2^ Manila Consulting Group McLean, VA United States

**Keywords:** MSM, gay, homosexual, bisexual, HIV, STD, Internet, survey, surveillance

## Abstract

**Background:**

Men who have sex with men (MSM) are disproportionately affected by human immunodeficiency virus (HIV) and there is evidence that this population is participating in increasingly risky sexual behavior. These changes are occurring in the context of new modes of online social interaction—many MSM now report first meeting their sex partners on the Internet. Better monitoring of key behavioral indicators among MSM requires the use of surveillance strategies that capitalize on these new modes of interaction. Therefore, we developed an annual cross-sectional behavioral survey of MSM in the United States, the American Men's Internet Survey (AMIS).

**Objective:**

The purpose of this paper was to provide a description of AMIS methods. In addition we report on the first cycle of data collection (December 2013 through May 2014; AMIS-2013) on the same key indicators used for national HIV behavioral surveillance.

**Methods:**

AMIS-2013 recruited MSM from a variety of websites using banner advertisements or email blasts. Adult men currently residing in the United States were eligible to participate if they had ever had sex with a man. We examined demographic and recruitment characteristics using multivariable regression modeling (*P*<.05) stratified by the participants' self-reported HIV status.

**Results:**

In the AMIS-2013 round, 79,635 persons landed on the study page and 14,899 were eligible, resulting in 10,377 completed surveys from MSM representing every US state. Participants were mainly white, 40 years or older, living in the US South, living in urban areas, and recruited from a general social networking website. Self-reported HIV prevalence was 10.73% (n=1113). Compared to HIV-negative/unknown status participants, HIV-positive participants were more likely to have had anal sex without a condom with any male partner in the past 12 months (72.24% versus 61.24%, respectively; *P*<.001) and more likely to have had anal sex without a condom with their last male sex partner who was discordant/unknown HIV status (42.95% versus 13.62%, respectively; *P*<.001). Illicit substance use in the past 12 months was more likely to be reported by HIV-positive participants than HIV-negative/unknown status participants (39.17% versus 26.85%, respectively; *P*<.001). The vast majority of HIV-negative/unknown status participants (84.05%) had been previously HIV tested, but less than half (44.20%) had been tested in the past 12 months. Participants 18-24 years of age were more likely than those 40 years or older to have had anal sex without a condom with a discordant/unknown HIV status partner, were more likely to report substance use, and were less likely to have been HIV tested. Compared to general social networking, those from a geospatial social networking website were more likely to have reported all risk behaviors but were more likely to have been HIV tested.

**Conclusions:**

The first round of AMIS generated useful behavioral measures from more than 10,000 MSM Internet users. Preliminary findings identified some subgroups of MSM Internet users that are at potentially higher risk of HIV acquisition/transmission. AMIS will provide an ongoing data source for examining trends in sexual risk behavior of MSM. This will help to plan and monitor the impact of programs to improve this population's health.

## Introduction

Men who have sex with men (MSM) continue to be disproportionately affected by human immunodeficiency virus (HIV). In the United States in 2012, more than 30,000 MSM were newly diagnosed with HIV infection, representing 66% of all diagnoses that year [1]. In contrast, gay/bisexual-identifying men account for <2% of the total US population [2]. There is also evidence that risky sexual behavior among MSM has increased in the past decade; data from the Centers for Disease Control and Prevention’s National HIV Behavioral Surveillance system (NHBS), that collects data on MSM in major US cities every three years, show a significant increase in the proportion of MSM who reported having anal sex without a condom between their 2005 and 2011 surveys [3]. From 2002-2011, MSM were also the only risk group for whom new HIV diagnoses did not decline [4], and HIV incidence among young MSM is estimated to have increased in recent years [5].

Contemporary to these increased HIV risks are new advances in HIV prevention for MSM. The past 5 years has seen new research proving the efficacy of antiretroviral medication to prevent HIV acquisition (pre-exposure prophylaxis or PrEP) and treatment of HIV positive persons that can reduce transmission [6,7]. Modeling has shown that implementing these biomedical interventions as part of an overall package of HIV prevention services could avert at least one quarter of HIV transmissions among MSM [8]. There are also now more sensitive tests that can detect HIV as early as 1 week after infection and a self-administered rapid HIV test [9,10].

All of these changes are occurring in a new context of social interaction. There are growing numbers of social networking website users and mobile application users [11]. MSM frequently report that they first met their sex partners online and spend considerable time looking for new partners this way [12-15]. This pattern of changing social context for MSM has been capitalized upon by many previous researchers who have successfully conducted entire cross-sectional research studies with MSM online [12-31].

There has also been progress made in large-scale behavioral surveys of MSM designed to monitor key risk behaviors over time. An example of such a system is the Gay Men's Sex Survey that has been conducted with Internet-recruited MSM in the United Kingdom every year since 2001 [32]. The Internet component of the survey now comprises the majority of the more than 10,000 annual survey respondents [33]. The largest ever Internet survey of MSM, the European MSM Internet Survey, was conducted in 2010 and collected data from 180,000 MSM in 38 European countries [34]. This study proved that the Internet is a viable and useful approach for large-scale behavioral surveillance.

In the United States, there has also been exploration of methods for routine monitoring of HIV-related risk behaviors among MSM. There was a one-time feasibility pilot of the Web-based HIV behavioral surveillance system (WHBS) conducted by the Centers for Disease Control and Prevention as supplement to NHBS [3,15,35]. The primary purpose of WHBS was to conduct behavioral surveillance with a standardized survey to compare to other data collections of MSM and estimate prevalence of risk behaviors among MSM Internet users. The pilot was successful at garnering a large sample of MSM.

There remains a need for establishing a system that can produce data for timely and large-scale monitoring of the behavior trends among MSM. In response to this need, we developed a new annual cross-sectional Internet survey of MSM in the United States, the American Men's Internet Survey (AMIS). The goal of AMIS is to collect surveys from 10,000 MSM each year in the United States in order to generate annual snapshots of relevant behaviors. In this paper, we provide the detailed description of our methods/materials, and report recruitment outcomes and some key indicators from our first round of data collection. To help with comparisons, the key indicators and the analytic approach were designed to mirror those used by NHBS’s most recent report on MSM risk behavior [3].

## Methods

### Recruitment and Enrollment

**Figure 1:**
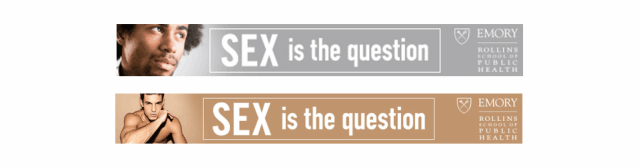
Example Banner Advertisements Used for the American Men’s Internet Survey, 2013.

AMIS participants were recruited through convenience sampling from a variety of websites using banner advertisements or email blasts to website members (hereafter referred to generically as "ads"). Ads depicted male models of various races and ethnicities (Figure 1). Men who clicked on the ads were taken directly to the survey website. Two survey platforms were used, depending on the recruitment website. Men recruited through ads posted to a geospatial social networking application were taken to our mobile-optimized survey hosted on a secure server administered by SurveyGizmo (Boulder, Colorado). Men recruited through ads posted elsewhere were taken to our survey hosted on a custom-designed survey website, also hosted on a secure server. Both survey websites used the same study content, used the same security standards, and were compliant with the Health Information Portability and Accountability Act.

The first page that men encountered on the study website contained a brief description of the study. Those who were interested in participating clicked a "begin survey" button that took them to the study's informed consent page which contained standard information regarding the study purpose, procedures, risks, benefits, protections, and investigator contact information. Those who consented to participate in the study were asked to check a box affirming this decision before continuing. Men who consented were then taken to a page with a brief eligibility screening questionnaire. To be eligible for the survey, participants had to be 18 years of age or older, consider themselves to be male, and report that they had oral or anal sex with a man at least once in the past. As is standard in behavioral research with MSM, transgender persons were excluded from the study because they are not MSM and recruitment approaches and behavioral risk measures should be specifically designed for this group. Persons who reported being <18 years of age or refused to provide their age were not asked any other screening questions. Persons who reported any gender identity other than male were not asked the sex behavior screening questions.

### Survey Administration

MSM who met the eligibility criteria started the online survey immediately. The survey consisted of a core questionnaire administered to all participants, 3 different subset questionnaires to which participants were randomized at the start of the survey, and an additional set of questions that were asked only of participants recruited through geospatial social networking ads. The subset questionnaires were of similar lengths. The intent of the randomization was to reduce overall survey response burden while still generating useful information on some additional behaviors. Participants were blind to this randomization and the randomized subset questions were interspersed with the core questions. The core questions were comprised of the following domains: demographics, sexual behavior, HIV testing history, drug and alcohol use, and HIV prevention services exposure. The randomized question subsets were comprised of the following domains: Subset A—knowledge and use of antiretrovirals for HIV prophylaxis and sexually transmitted disease testing/vaccination; Subset B—disclosure of sexual identity and experiences of stigma; and Subset C—additional details about most recent male sex partner. The participants recruited from the geospatial social networking website received an additional set of questions about a potential mobile HIV prevention app and about acute HIV infection. The core and subset questions were derived from those validated and used by NHBS and used the same timeframes for behaviors [3,36]. The full questionnaire is presented in Multimedia Appendix 1.

To maximize the efficiency of the survey, questions were presented adaptively using a participant's previous responses to determine the path of questioning or auto-filling responses into the text of follow-up questions. On average there were 5 questions per survey webpage. Participants could decline to answer any question. Participants were not asked to correct, verify, or complete responses at the end of their survey. A participant who left their survey would not be able to see any of their previous responses on returning and would have to start the whole survey over again. We did not use cookies to minimize information on a participant's computer that could potentially identify them as an AMIS participant. We did collect Internet protocol (IP) address to allow us to determine residency and identify potential duplicate respondents.

### Human Subjects Protections

The study was conducted in compliance with federal regulations governing protection of human subjects and was reviewed and approved by our institution's human subjects research review board. No incentive was provided to the participants. Datasets for analyses are stored on secure data servers with access only granted to study staff. The study data are protected under a federal certificate of confidentiality that prevents legal action to force data release.

### Measures and Analyses

Recruitment outcomes for the study are reported as screening, eligibility, unduplicated responses, survey success and reporting sex with a man in the past 12 months. Screening was defined as those who started the screening questionnaire. Overall survey eligibility and individual criteria for ineligibility are presented and were based on survey responses for age, gender and sexual behavior. US residency was determined by either a response of a valid US ZIP code of residence or, for those with no valid ZIP code response, an IP address assigned to a location in the US. Unduplicated responses were determined based on the de-duplication algorithm using IP address, response matching and survey success (see Multimedia Appendix 2 for details). If an observation had a missing value for the first question of at least two consecutive sections, their response was considered incomplete and was not included in the final dataset. All other surveys were considered a "success". Sex with a man in the past 12 months was determined by reporting of one or more partners in response to the question, "In the past 12 months, with how many different men have you had oral or anal sex?"

In addition to standard individual demographic characteristics, we categorized participants based on recruitment source, self-reported HIV status, and geography. The embedded links in the ads were unique and allowed us to determine from which website participants were recruited. We categorized these based on target audience and purpose: gay social networking (n=2), gay general interest (n=3), general social networking (n=1), and geospatial social networking (n=1). We do not provide the names of the websites to preserve operator/client privacy, particularly where a website category has only one operator. Gay social networking websites are those designed for gay or bisexual men to connect with one another, including those attempting to connect for sex. Gay general interest websites are those designed specifically for gay or bisexual men's general interests, such as news stories, public policy advocacy, and travel. The general social networking website is one designed for the general public to connect with others and is not specifically focused on connecting sexual partners. The geospatial social networking website runs on smart cellular telephones and is designed for gay and bisexual men to connect to other men who are near their current location, including those attempting to connect for sex. Self-reported HIV status was determined from responses to questions about having ever had an HIV test, results of the most recent HIV test, and having ever had a positive HIV test. Participants were categorized as HIV-positive, HIV-negative, or unknown status.

We used a combination of county and ZIP code of residence to determine state, US Census-based region, NHBS city residency, and population density. Cities included in the NHBS as of 2011 were as follows: Atlanta, Georgia; Baltimore, Maryland; Boston, Massachusetts; Chicago, Illinois; Denver, Colorado; Houston, Texas; Los Angeles, California; Miami, Florida; Newark, New Jersey; New York City, New York; Philadelphia, Pennsylvania; San Diego, California; San Francisco, California; San Juan, Puerto Rico; Washington, DC; Dallas, Texas; Detroit, Michigan; New Orleans, Louisiana; Nassau-Suffolk, New York; and Seattle, Washington.

The participants who were eligible, unduplicated, successful, and reported male-male sex in the past 12 months were included in analyses of participant characteristics and behavior. Overall chi-square tests were used to identify whether participant characteristics significantly differed between recruitment website types and between question subsets. Following the format used by NHBS in the most recent report of MSM behaviors [3], the prevalence of sex and substance-using behaviors were stratified by self-reported HIV status as either HIV-positive or HIV-negative/unknown. Sexual behaviors were assessed with male partners for either the past 12 months (anal intercourse without a condom with any partner) or for the last partner (anal intercourse without a condom with a discordant or unknown status partner) [3]. HIV serostatus discordance was based on the participant’s HIV status and the status of their sex partner. Discordance was defined as either the participant or partner having unknown status or when one was HIV-negative and the other was HIV-positive. Sexually transmitted infection (STI) testing and diagnosis in the past 12 months was only assessed for one-third of randomized participants and included gonorrhea, Chlamydia and syphilis [37]. Illicit substance use in the past 12 months was assessed as the use of any type of illicit substance by any means of delivery, including injection [37]. Binge alcohol drinking in the past 12 months was assessed as having at least once had 5 or more alcoholic drinks in one sitting [38].

Prevalence of sexual behaviors with male partners, substance use and HIV testing were also presented by race/ethnicity, age group, NHBS city residency, and website recruitment type within the HIV status categories. To determine whether there were significant differences in reported behaviors of different participant subgroups, we conducted multivariable modeling stratified by self-reported HIV status in which each behavior was modeled as the dependent variable and including the following independent variables: race/ethnicity, age group, NHBS city residency, and recruitment website type. We also conducted multivariable logistic regression modeling to determine significant differences in behaviors based on self-reported HIV status while controlling for race/ethnicity, age group, NHBS city residency, and recruitment website type. HIV testing behaviors were only examined among those who did not report that they were HIV-positive and were also presented by participant characteristics. Multivariable logistic regression results are presented as Wald chi-square *P* values to denote an independently significant difference in the behavior for each sub-group compared to a referent group. Statistical significance was determined at *P*<.05.

## Results

### Recruitment, Enrollment, and Survey Completion

The 2013 data collection round of AMIS (AMIS-2013) ran from December 2013 through May 2014, and resulted in 79,635 persons clicking on the ads and landing on the study's recruitment page (Table 1). Most were from a general social networking website (36,281/79,635, 45.56%) or a geospatial social networking website (27,720/79,635, 34.81%). About a quarter of those who landed on the study's page (18,669/79,635, 23.44%) consented to take part in it. The proportion providing consent varied by recruitment website, with the highest proportion consenting among those recruited from gay general interest websites (36.97%) and the lowest proportion among recruits from the geospatial social networking website (14.18%). Most who were screened were eligible (79.81%). The most common reasons for ineligibility were not being male or reporting not having male-male sex. This was true even of the websites that were specifically marketed to gay persons.

There were 709 (4.76%) surveys determined to likely be from duplicate participants. Among unduplicated surveys, most were considered successful (12,369/14,190, 87.17%). Most successful surveys were among men who reported having sex with another man in the past 12 months (10,377/12,369, 83.90%). The median duration of completion for successful surveys from MSM participants was 14 minutes. AMIS-2013 was managed, implemented, and analyzed by 4 part-time staff (2 faculty, 1 post-doctoral fellow, and 1 program associate). The total cost to implement the survey was approximately 150,000 USD or 15 USD per successful survey.

**Table 1:** Recruitment outcomes with different recruitment website types for the American Men’s Internet Survey, United States, 2013.

Recruitment outcomes		Total		Gay social networking		General gay interest		General social networking		Geospatial social networking	
				(n=2)		(n=3)		(n=1)		(n=1)	
		n	(%)	n	(%)	n	(%)	n	(%)	n	(%)
Clicked ad		79,635		6889		8745		36,281		27,720	
Consented^a^		18,669	(23.44)	1404	(23.38)	3233	(36.97)	10,100	(27.84)	3932	(14.18)
**Ineligible** ^b^		3770		223	(15.88)	632	(19.55)	2304	(22.81)	611	(15.54)
	Not 18+ years of age^c^	636	(16.87)	54	(24.22)	109	(17.25)	311	(13.50)	162	(26.51)
	Not male^c^	2132	(56.55)	175	(78.48)	450	(71.20)	1052	(45.66)	455	(74.47)
	Not ever MSM^c^	3628	(96.23)	223	(100)	631	(99.84)	2304	(100.00)	470	(76.92)
	Not a resident^c^	1408	(37.35)	119	(53.36)	369	(58.39)	732	(31.77)	188	(30.77)
Eligible^b^		14,899	(79.81)	1181	(84.12)	2601	(80.45)	7796	(77.19)	3321	(84.46)
Unduplicated^d^		14,190	(95.24)	1130	(95.68)	2516	(96.73)	7373	(94.57)	3171	(95.48)
Success^e^		12,369	(87.17)	987	(87.35)	2270	(90.22)	6735	(91.35)	2377	(74.96)
MSM^f^ past 12 months^g^		10,377	(83.90)	802	(81.26)	1958	(86.26)	5336	(79.23)	2281	(95.96)

^a^ Proportion is of total who clicked ad

^b^ Proportion is among consented

^c^ Proportion is among total ineligible

^d^ Proportion is among eligible. Unduplicated removes participants who were marked as duplicates using IP address and demographic data.

^e^ Proportion is among unduplicated. Success removes participants who did not pass the survival analysis test for survey completeness.

^f^ MSM: Men who have sex with men

^g^ Proportion is among successes

### Participant Characteristics

**Figure 2:**
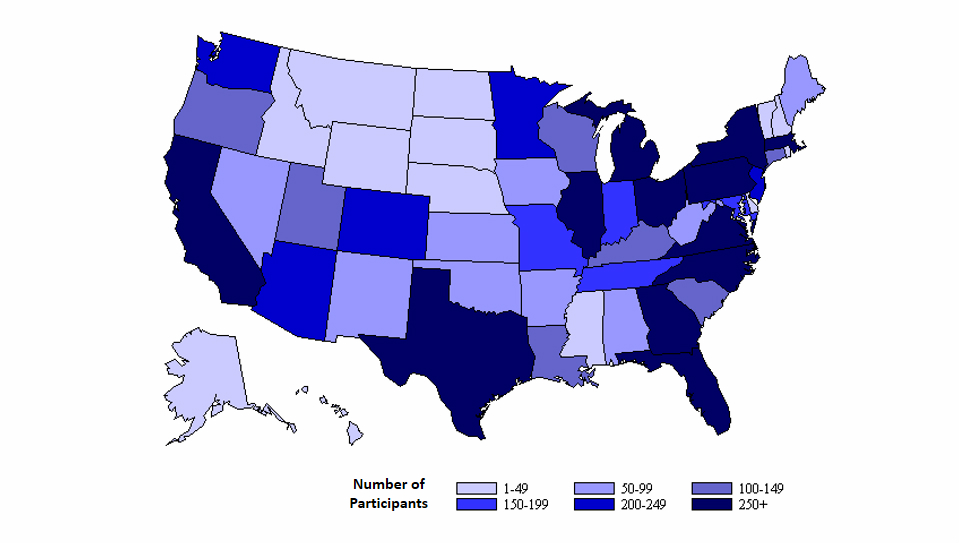
Number of MSM Participants in the American Men’s Internet Survey by State, 2013.

Of the 10, 377 participants in AMIS-2013 who had a successful survey and had male-male sex in the past 12 months, more than three-quarters were white, non-Hispanic (Table 2). Nearly half of the participants were ≥ 40 years of age; others were distributed almost equally between younger age groups. The most common region of residence was the South followed by the West. AMIS-2013 had participants from all US states and at least 100 participants from each of 27 states (Figure 2). There were approximately twice as many participants from urban areas as there were from rural areas, and about one-third of participants lived in NHBS cities. Overall, 1113 (10.73%) participants reported being HIV positive and 9264 (89.27%) reported being HIV negative or having an unknown HIV serostatus. Most participants were recruited from a general social networking website. The second most common recruitment site was the geospatial social networking website.

There were significant differences in participant characteristics based on where they were recruited (Table 2, all *P*<.001). Most of those differences were observed among participants recruited from the geospatial social networking website, who were less likely be white, less likely be 40 years or older, less likely to live in an NHBS city, more likely to live in the South, more likely to live in urban areas, and more likely to report being HIV positive. There were no significant differences in the characteristics of survey sub-samples that received the 3 different randomized questionnaires (see Multimedia Appendix 3).

**Table 2:** Characteristics of MSM participants in the American Men's Internet Survey by recruitment website type, United States, 2013.

Participant characteristics		Total		Gay social networking		General gay interest		General social networking		Geospatial social networking	
				(n=2)		(n=3)		(n=1)		(n=1)	
		n	(%)	n	(%)	n	(%)	n	(%)	n	(%)
**Race/Ethnicity**											
	Black, non-Hispanic	354	(3.41)	37	(4.61)	48	(2.45)	119	(2.23)	150	(6.58)
	Hispanic	1084	(10.45)	66	(8.23)	127	(6.49)	417	(7.81)	474	(20.78)
	White, non-Hispanic	8076	(77.83)	645	(80.42)	1638	(83.66)	4351	(81.54)	1442	(63.22)
	Other or multiple races	863	(8.32)	54	(6.73)	145	(7.41)	449	(8.41)	215	(9.43)
**Age (years)**											
	18-24	1982	(19.10)	96	(11.97)	246	(12.56)	1067	(20.00)	573	(25.12)
	25-29	1515	(14.60)	76	(9.48)	256	(13.07)	693	(12.99)	490	(21.48)
	30-39	1918	(18.48)	80	(9.98)	398	(20.33)	881	(16.51)	559	(24.51)
	40 or older	4962	(47.82)	550	(68.58)	1058	(54.03)	2695	(50.51)	659	(28.89)
**Region**											
	Midwest	2078	(20.03)	203	(25.31)	347	(17.72)	1147	(21.50)	381	(16.70)
	Northeast	2050	(19.76)	176	(21.95)	444	(22.68)	1119	(20.97)	311	(13.63)
	South	3558	(34.29)	279	(34.79)	648	(33.09)	1681	(31.50)	950	(41.65)
	West	2503	(24.12)	144	(17.96)	518	(26.46)	1382	(25.90)	459	(20.12)
	US dependent areas	8	(0.08)	0	(0.00)	1	(0.05)	7	(0.13)	0	(0.00)
**NHBS^a^ City Resident**											
	Yes	3268	(31.49)	244	(30.42)	858	(43.82)	1750	(32.80)	416	(18.24)
	No	7109	(68.51)	558	(69.58)	1100	(56.18)	3586	(67.20)	1865	(81.76)
**Population Density**											
	Rural	3833	(36.94)	360	(44.89)	626	(31.97)	2129	(39.90)	718	(31.48)
	Urban	6544	(63.06)	442	(55.11)	1332	(68.03)	3207	(60.10)	1563	(68.52)
**Self-reported HIV Status**											
	Positive	1113	(10.73)	50	(6.23)	155	(7.92)	439	(8.23)	469	(20.56)
	Negative	7657	(73.79)	535	(66.71)	1556	(79.47)	4018	(75.30)	1548	(67.86)
	Unknown	1607	(15.49)	217	(27.06)	247	(12.61)	879	(16.47)	264	(11.57)
**Total**		10,377		802		1958		5336		2281	

^a^ NHBS: National HIV Behavioral Surveillance System

### Sexual Behaviors

Most participants had anal sex without a condom with another man in the past 12 months (Table 3). The proportion who had anal sex without a condom was significantly higher among HIV-positive participants compared to HIV-negative/unknown status participants (72.24% versus 61.24%, respectively; *P*<.001). Compared to HIV-negative/unknown status participants, a larger proportion of HIV-positive participants had anal sex without a condom with their last male sex partner who was discordant/unknown status (13.62% versus 42.95%, respectively; *P*<.001).

Among those who were HIV-positive, Hispanic participants were less likely than white participants to report anal sex without a condom in the past 12 months and black participants were less likely than white participants to report anal sex without a condom with an HIV-negative/unknown status partner (Table 3). Participants 18-24 years of age were more likely to report anal sex without a condom with an HIV-negative/unknown status partner compared to participants ≥40 years of age. Nearly two-thirds of HIV-positive participants 18-24 years reported anal sex without a condom with a partner who was either HIV-negative or of unknown status. HIV-positive participants who lived in NHBS cities were also more likely than those living elsewhere to report anal sex without a condom in the past 12 months. Compared to HIV-positive participants from a general social networking website, those recruited from a geospatial social networking website were also significantly more likely to report anal sex without a condom and anal sex without a condom with an HIV-negative or unknown status partner.

Among those who were HIV-negative or unknown status, those 25-39 years of age were significantly more likely to report anal sex without a condom compared to participants ≥40 years of age (Table 3). Participants 18-24 years of age were more likely to report anal sex without a condom with an HIV-positive or unknown status partner compared to participants ≥ 40 years of age. Compared to participants recruited from the general social networking website, those from other websites had significant differences sexual behaviors. Participants from gay social networking websites were less likely to report anal sex without a condom, but those from a geospatial social networking website were more likely to report this behavior. Participants from gay social networking and geospatial social networking websites were more likely to report anal sex without a condom with an HIV-positive/unknown status partner.

Among MSM participants who were HIV-positive, 3.05% (34/1113) also had sex with a woman and 1.17% (13/1113) of those participants reported vaginal sex without a condom in the past 12 months (data not presented in a table). Among those who were HIV-negative or unknown status, 10.29% (953/9264) also had sex with a woman and 6.50% (602/9264) had vaginal sex without a condom in the past 12 months. Both behaviors were significantly more likely among HIV-negative or unknown status participants than among HIV-positive participants (both *P*<.001).

**Table 3:** Sexual Behaviors with Male Partners of MSM Participants in the American Men's Internet Survey, United States, 2013.

Participant characteristics			n in sample	Anal intercourse without a condom in the past 12 months			Anal intercourse without a condom with last sex partner of discordant or unknown HIV status		
				n	(%)	*P* value^a^	n	(%)	*P* value^a^
**HIV-positive overall**			1113	804	(72.24)	<.001^b^	478	(42.95)	<.001^b^
	**Race/Ethnicity**								
		Black, non-Hispanic	63	46	(73.02)	.580	21	(33.33)	.009
		Hispanic	162	110	(67.90)	.032	74	(45.68)	.780
		White, non-Hispanic	810	590	(72.84)	REF	343	(42.35)	REF
		Other or multiple races	78	58	(74.36)	.335	40	(51.28)	.027
	**Age (years)**								
		18-24	90	78	(86.67)	.060	56	(62.22)	.033
		25-29	124	104	(83.87)	.085	66	(53.23)	.504
		30-39	201	148	(73.63)	.041	94	(46.77)	.244
		40 or older	698	474	(67.91)	REF	262	(37.54)	REF
	**NHBS city resident^c^**								
		Yes	311	232	(74.60)	.016	119	(38.26)	.793
		No	802	572	(71.32)	REF	359	(44.76)	REF
	**Recruitment website type**								
		Gay social networking	50	35	(70.00)	.987	25	(50.00)	.122
		General gay interest	155	103	(66.45)	.139	57	(36.77)	.108
		General social networking	439	291	(66.29)	REF	139	(31.66)	REF
		Geospatial social networking	469	375	(79.96)	.001	257	(54.80)	.002
**HIV-negative or unknown overall**			9264	5673	(61.24)	REF	1262	(13.62)	REF
	**Race/Ethnicity**								
		Black, non-Hispanic	291	179	(61.51)	.297	55	(18.90)	.578
		Hispanic	922	592	(64.21)	.934	183	(19.85)	.125
		White, non-Hispanic	7266	4408	(60.67)	REF	901	(12.40)	REF
		Other or multiple races	785	494	(62.93)	.651	123	(15.67)	.998
	**Age (years)**								
		18-24	1892	1217	(64.32)	.813	323	(17.07)	.001
		25-29	1391	937	(67.36)	.019	193	(13.87)	.157
		30-39	1717	1218	(70.94)	<.001	241	(14.04)	.613
		40 or older	4264	2301	(53.96)	REF	505	(11.84)	REF
	**NHBS city resident^c^**								
		Yes	2957	1784	(60.33)	.596	370	(12.51)	.737
		No	6307	3889	(61.66)	REF	892	(14.14)	REF
	**Recruitment website type**								
		Gay social networking	752	365	(48.54)	<.001	95	(12.63)	.275
		General gay interest	1803	1113	(61.73)	.134	205	(11.37)	.002
		General social networking	4897	2926	(59.75)	REF	533	(10.88)	REF
		Geospatial social networking	1812	1269	(70.03)	<.001	429	(23.68)	<.001

^a^ Wald chi-square from multivariable logistic regression comparing behavior (yes versus no) among group with some characteristic compared to a referent (REF) group.

^b^ Wald chi-square from multivariable logistic regression comparing behavior (yes versus no) among HIV-positive participants compared to HIV-negative or unknown serostatus particiants. Model controlled for race/ethnicity, age, NHBS residency, and website type.

^c^ NHBS = National HIV Behavioral Surveillance System

### Substance Use Behaviors

Illicit substance use in the past 12 months was more likely to be reported by HIV-positive participants than HIV-negative/unknown status participants (39.17% versus 26.85%, respectively; *P*<.001; Table 4). Approximately half of participants reported binge drinking alcohol in the past 12 months, and there was no difference by participants' HIV status (55.53% for HIV-positive and 58.27% for HIV-negative/unknown; *P*=.681).

Among participants who were HIV-positive, those 25-29 years of age were more likely to report using illicit drugs and binge drank alcohol compared with those ≥40 years of age (Table 4). More than half of those 25-29 years of age reported using illicit substances and more than three-quarters reported binge drinking alcohol in the past 12 months. Compared to participants recruited from a general social networking website, those from gay general interest websites were less likely to report binge drank alcohol.

Among participants who were HIV-negative or unknown status, Hispanic participants were more likely and black or other/multiracial participants were less likely than white participants to report binge drinking alcohol (Table 4). Compared to participants ≥40 years of age, those 18-29 were more likely to report using illicit substances and binge drinking alcohol. Approximately one-third of these younger participants reported using illicit substances and three-quarters reported binge drinking alcohol in the past 12 months. Participants who resided in NHBS cities were also more likely to report using illicit substances and binge drinking. Compared to participants from the general social networking website, those from gay social networking websites were less likely to report substance use and those from a geospatial social networking website were more likely to report substance use.

**Table 4:** Substance using behaviors of MSM participants in the American Men's Internet Survey, United States, 2013.

				Substance use behaviors in the past 12 months					
Participant characteristics			n in sample	Used illicit drug			Binge drank alcohol		
				n	(%)	*P* value^a^	n	(%)	*P* value^a^
**HIV-positive overall**			1113	436	(39.17)	<.001^b^	618	(55.53)	.681^b^
	**Race/Ethnicity**								
		Black, non-Hispanic	63	27	(42.86)	.722	42	(66.67)	.434
		Hispanic	162	68	(41.98)	.529	102	(62.96)	.981
		White, non-Hispanic	810	304	(37.53)	REF	430	(53.09)	REF
		Other or multiple races	78	37	(47.44)	.210	44	(56.41)	.435
	**Age (years)**								
		18-24	90	37	(41.11)	.360	66	(73.33)	.195
		25-29	124	67	(54.03)	.007	100	(80.65)	<.001
		30-39	201	94	(46.77)	.404	132	(65.67)	.502
		40 or older	698	238	(34.10)	REF	320	(45.85)	REF
	**NHBS city resident**								
		Yes	311	131	(42.12)	.050	163	(52.41)	.625
		No	802	305	(38.03)	REF	455	(56.73)	REF
	**Recruitment website type**								
		Gay social networking	50	15	(30.00)	.309	18	(36.00)	.025
		General gay interest	155	57	(36.77)	.809	78	(50.32)	.271
		General social networking	439	159	(36.22)	REF	227	(51.71)	REF
		Geospatial social networking	469	205	(43.71)	.121	295	(62.90)	.131
**HIV-negative or unknown overall**			9264	2487	(26.85)	REF	5398	(58.27)	REF
	**Race/Ethnicity**								
		Black, non-Hispanic	291	75	(25.77)	.075	165	(56.70)	.007
		Hispanic	922	275	(29.83)	.960	612	(66.38)	.007
		White, non-Hispanic	7266	1912	(26.31)	REF	4187	(57.62)	REF
		Other or multiple races	785	225	(28.66)	.336	434	(55.29)	.018
	**Age (years)**								
		18-24	1892	686	(36.26)	<.001	1349	(71.30)	<.001
		25-29	1391	452	(32.49)	.006	1069	(76.85)	<.001
		30-39	1717	521	(30.34)	.466	1143	(66.57)	.326
		40 or older	4264	828	(19.42)	REF	1837	(43.08)	REF
	**NHBS** ^c^ **city resident**								
		Yes	2957	828	(28.00)	.002	1788	(60.47)	<.001
		No	6307	1659	(26.30)	REF	3610	(57.24)	REF
	**Recruitment website type**								
		Gay social networking	752	118	(15.69)	<.001	339	(45.08)	<.001
		General gay interest	1803	474	(26.29)	.176	1033	(57.29)	.773
		General social networking	4897	1255	(25.63)	REF	2773	(56.63)	REF
		Geospatial social networking	1812	640	(35.32)	<.001	1253	(69.15)	<.001

^a^ Wald chi-square from multivariable logistic regression comparing behavior (yes versus no) among group with some characteristic compared to a referent (REF) group.

^b^ Wald chi-square from multivariable logistic regression comparing behavior (yes versus no) among HIV-positive participants compared to HIV-negative or unknown serostatus particiants. Model controlled for race/ethnicity, age, NHBS residency, and website type.

^c^ NHBS = National HIV Behavioral Surveillance System

### HIV and STI Testing Behaviors

HIV testing behaviors were only examined among those who did not report being HIV-positive. Most of those participants (84.05%) had been previously tested for HIV infection, but less than half (44.20%) reported being tested in the past 12 months (Table 5). Compared to white participants, black participants were more likely to report ever having been tested. Compared to participants ≥40 years of age, those 18-24 years were less likely to report ever having been tested or having been tested in the past 12 months. Those 30-39 years were more likely to have been tested ever or in the past 12 months. Compared to participants recruited from the general social networking website, those from other websites had significant differences in reported HIV testing behaviors. Participants from gay social networking websites were less likely to report having been tested ever or in the past 12 months. Participants from general gay interest websites and from a geospatial social networking website were more likely to report having been tested ever or in the past 12 months.

Among participants who were HIV-positive and got the randomized STI testing and diagnosis questions, 56.54% (216/382) had an STI test in the past 12 months and 19.89% (76/382) had any STI diagnosis: 9.16% (35/382) were diagnosed with gonorrhea, 7.07% (27/382) with Chlamydia and 9.69% (37/382) with syphilis. Among participants who were HIV-negative or unknown status, 24.48% (758/3096) had an STI test in the past 12 months and 4.98% (154/3096) had any STI diagnosis: 2.68% (83/3096) were diagnosed with gonorrhea, 2.62% (81/3096) with Chlamydia and 1.26% (39/3096) with syphilis. Compared to participants who were HIV-negative or of unknown status, those who were HIV-positive were significantly more likely to have been tested for and to have had any diagnosis of an STI (both *P*<.001).

**Table 5:** HIV testing behaviors of HIV-negative or unknown status MSM participants in the American Men's Internet Survey, United States, 2013.

			Testing behaviors					
Participant characteristics		n in sample	HIV tested ever			HIV tested past 12 months		
			n	(%)	*P* value^a^	n	(%)	*P* value^a^
**Race/Ethnicity**								
	Black, non-Hispanic	291	256	(87.97)	.009	160	(54.98)	.133
	Hispanic	922	763	(82.75)	.541	458	(49.67)	.285
	White, non-Hispanic	7266	6117	(84.19)	REF	3073	(42.29)	REF
	Other or multiple races	785	649	(82.68)	.146	374	(47.64)	.786
**Age (years)**								
	18-24	1892	1224	(64.69)	<.001	815	(43.08)	<.001
	25-29	1391	1176	(84.54)	.295	688	(49.46)	.076
	30-39	1717	1571	(91.50)	<.001	878	(51.14)	<.001
	40 or older	4264	3814	(89.45)	REF	1684	(39.49)	REF
								
**NHBS** ^b^ **city resident**								
	Yes	2957	2599	(87.89)	<.001	1403	(47.45)	<.001
	No	6307	5186	(82.23)	REF	2662	(42.21)	REF
**Recruitment website type**								
	Gay social networking	752	542	(72.07)	<.001	236	(31.38)	<.001
	General gay interest	1803	1570	(87.08)	.008	730	(40.49)	<.001
	General social networking	4897	4084	(83.40)	REF	1933	(39.47)	REF
	Geospatial social networking	1812	1589	(87.69)	<.001	1166	(64.35)	<.001
Total		9264	7786	(84.05)		4095	(44.20)	

^a^ Wald chi-square from multivariable logistic regression comparing behavior (yes versus no) among group with some characteristic compared to a referent (REF) group.

^b^ NHBS = National HIV Behavioral Surveillance System

## Discussion

### Principal Findings

The first round of data collection for AMIS was successfully implemented and resulted in more than 10,000 surveys from a diverse sample of Internet using MSM residing in all US states. There were notable differences in key behavioral indicators sorted by recruitment website type. In particular, the geospatial social networking website produced a sample made up of participants with significantly different demographic characteristics and self-reported HIV status. Participants recruited from that site were also substantially more risky but also more likely to have been HIV tested. Future samples for AMIS and other analyses with this data will have to take these differences into consideration in study and analysis design.

One purpose of AMIS was to generate useful annual behavioral data to compare to NHBS-MSM which is only conducted every 3 years [3]. Understanding the differences between MSM recruited in the “in-person” NHBS surveys and our Internet-recruited surveys will allow correlation of NHBS and AMIS results and evaluation of trends in years between NHBS surveys. Compared to the most recent NHBS-MSM data from 2011, our study found a higher prevalence of all of the assessed risk behaviors, including for our sub-sample that lived in NHBS cities. Data from our study do not explain this difference, because where the few significant differences exist, AMIS participants that lived in NHBS cities were more likely than those that lived elsewhere to report risky behaviors. This risk difference between the two samples could be partially explained by differences between the demographic composition of the AMIS and NHBS samples, where NHBS had more participants who were younger and black. Our own study has shown that older participants were less likely to report risky behavior and other research has shown black MSM tend to report less unprotected sex and drug use [39]. Demographic differences between online and real-world samples have also been previously reported even where the geographic areas are the same, though unlike our study, most have found Internet surveys to have a higher proportion of younger participants than their comparison surveys [15,18,40]. The demographic characteristics of the AMIS sample and the WHBS pilot sample were more similar to one another than to NHBS also indicating that these differences in behaviors may be due less to geographic differences and more to sampling approach [35].

Regardless of those differences, our findings emphasize how annual AMIS data may complement those from the 3-year NHBS samples, providing timely and useful information for prevention program planning for MSM in many US states. In addition, the complementary data from AMIS may come at considerably reduced cost compared to the venue-recruited NHBS sample. The entire AMIS data collection and analysis support costs approximately $15 per survey whereas NHBS costs at least $1000 per survey based only on federal funding to local jurisdictions and not including CDC management or analysis costs [41]. NHBS is rightfully more expensive than AMIS because it involves a more comprehensive and detailed survey approach that requires full survey teams in each city to conduct the in-person method, participant incentives, and laboratory expenses for rapid HIV testing.

Substantial proportions of AMIS participants reported sexual behaviors that may potentially pose a risk of HIV transmission. Over 40% of HIV-positive participants had anal sex without a condom with a potentially serodiscordant male partner. More than half of HIV-negative/unknown status participants also had anal sex without a condom, though most reported that they perceived their partners to be HIV-negative. Serostatus discussions between sex partners are an important part of HIV prevention, but previous studies have shown that those discussions may be based on inaccurate information because of high rates of undiagnosed HIV infection among MSM [39,42-44]. HIV-positive persons who are taking antiretroviral medications and have their HIV virus suppressed are also significantly less likely to transmit HIV to their sex partners, but we do not have this information for our participants [7]. Younger MSM were also significantly more likely to have had anal sex without a condom, a pattern also seen in the NHBS data [3]. For the youngest group in our study, 18-24 years of age, this is combined with a significantly increased likelihood that they are substance users and a reduced likelihood that they have ever been HIV tested. This presents a potentially heightened risk for HIV transmission in this group.

Compared to AMIS, NHBS had similar proportions who reported anal sex without a condom, but NHBS reported a substantially lower proportion of HIV-positive participants who had serodiscordant anal sex without a condom than our study (13% versus 43%, respectively) [3]. This may be explained by other differences in the samples/procedures (eg, demographics, self- versus interviewer-administered survey) or may be due to some fundamental difference in how sexual encounters are negotiated by HIV-positive MSM who were recruited from the internet. This indicator did not substantially differ between the HIV-negative/unknown status participants in the two studies. Similar to our findings, the EMIS study also found a significantly higher risk of non-concordant unprotected anal intercourse among HIV-positive participants compared to HIV-negative participants, but reported a substantially higher proportion of their participants overall had engaged in this behavior compared to our study (30% versus 17%, respectively) [34]. The timeframes for this behavior between our study and EMIS were not the same and may explain at least part of this difference; we examined behavior with last male sex partner and EMIS examined behavior with any male sex partner in the past 12 months. Collaborations should be explored to allow comparisons of non-concordant anal intercourse without condoms between these studies.

### Limitations

Several limitations to the AMIS methods and these analyses should be noted. First, AMIS data are not generalizable to all MSM in the US or to all MSM online. AMIS used a convenience sampling approach online and we cannot determine the degree or direction of response bias. Though we included several different types of websites to increase sample diversity, the websites still represent a small fraction of those that MSM likely use. Second, there was under-representation of black or African American MSM in the AMIS sample, a problem common to Internet research [23]. This group is disproportionately impacted by HIV infection and the small AMIS sample size limits our ability to do more detailed analyses with these data. Third, the survey only involved self-report of behaviors. Though anonymous self-administered surveys such as AMIS may be less prone to obsequiousness bias [45-47], it is possible that less socially desirable responses may be under-reported (eg, anal sex without a condom) and more socially desirable responses may be over-reported (eg, recent HIV testing). Finally, the analyses presented here were only preliminary to illustrate the success of the AMIS method in generating key behavioral indicators. Although we report statistical tests in our behavioral analyses that controlled for some demographic characteristics, there were relatively few factors in the model which may not have resolved all confounding. Therefore interpretation regarding the independence of statistical relationships should be made with caution until more detailed modeling can be conducted and reported.

### Future Directions

We are nearing completion of our second round of data collection of 10,000 surveys and intend to conduct the third round in the summer of 2015. The data we have collected to-date have been shared with state health departments in standardized reports to enable better planning for public health interventions (see Multimedia Appendix 4 for an example report). We have also made individual state AMIS datasets available to each state's public health authorities so they can conduct further analyses for their own MSM residents. We have developed and deployed what will eventually become the largest ongoing Internet survey of MSM in the United States, and we envision that AMIS will become a useful tool in our joint endeavors to improve the health and wellbeing of this population.

## Appendix

Multimedia Appendix 1 AMIS 2013 questionnaire.

Multimedia Appendix 2 AMIS deduplication algorithm.

Multimedia Appendix 3 Characteristics of MSM participants in the American Men's Internet Survey by randomization to different question subsets, United States, 2013.

Multimedia Appendix 4 AMIS 2013 sample state report.

## Abbreviations

AMIS: American Men’s Internet Survey
CDC: Centers for Disease Control and Prevention
EMIS: European MSM Internet Survey
HIV: human immunodeficiency virus
IP: Internet protocol
MSM: men who have sex with men
NHBS: National HIV Behavioral Surveillance System
STI: sexually transmitted infection
WHBS: Web-based HIV Behavioral Surveillance System
