# Supplementary material for: The Annual American Men's Internet Survey of Behaviors of Men Who Have Sex With Men in the United States: Protocol and Key Indicators Report 2013
Source: JMIR Public Health Surveill. 2015 Apr 17;1(1):e3. doi: 10.2196/publichealth.4314 (PMC4869242; doi:10.2196/publichealth.4314)
Supplement: Multimedia Appendix 1 [file publichealth_v1i1e3_app1.pdf]

American Men's Internet Survey, 2013

Survey Domains **Page Number**

Consent....1

Eligibility....3

Demographics....5

Sexual Behavior with Females in the Past 12 Months....9

Sexual Behavior with Males in the Past 12 Months....11

Sexual Behavior with Last Male Partner....15

Alcohol Use....24

Injection Drug Use....26

Non-injection Drug Use....28

HIV Testing....30

PrEP and nPEP....34

Acute Infection....37

Stigma and Discrimination....40

Hepatitis and STIs....43

Prevention Activities....47

Join over 10,000 men in the U.S. by taking this sexual health survey!

We all know how to keep ourselves safe. Yet, guys continue to test positive. We need your help to figure out why. Your confidential answers will be pooled with tens of thousands more. The resulting data will help researchers better understand patterns of behavior- both sexual and health promoting- among men in our communities, and to make sure our prevention resources have the greatest impact.

Thank you for your interest in our survey. Please note:

1. Your answers are confidential: we don't have any information about who you are beyond the questions you answer.

2. This survey includes some personal questions. You can choose to not answer any questions that make you feel uncomfortable.

3. If you agree to be in this study, you will first be screened to see if you qualify to be in the study. If you qualify, you will be given the opportunity to complete the survey.

If you have any questions or comments you may contact the research staff at sexisthequestion@emory.edu.

Please read the information below about the study, and indicate whether you consent to participate in the study. *

( ) I consent to participate in the survey.

( ) I do not consent to participate in the survey.

Click here for a printable version of the consent form.

Consent form displayed here.

How old are you?

____

Eligibility Screener

During 2013 or 2014, did you already complete at least part of Sex is the Question?

( ) No

( ) Yes

( ) I prefer not to answer

( ) Don't know

Do you consider yourself to be Hispanic or Latino?

( ) No

( ) Yes

( ) I prefer not to answer

( ) Don't know

Which racial group or groups do you consider yourself to be in? Check all that apply.

[ ] American Indian or Alaska Native

[ ] Asian

[ ] Black or African American

[ ] Native Hawaiian or Other Pacific Islander

[ ] White

[ ] I prefer not to answer

[ ] Does not apply

[ ] Don't know

What zipcode do you live in?

____

Do you consider yourself to be male, female, or transgender?

( ) Male

( ) Female

( ) Transgender

( ) Don't know

( ) Prefer not to answer

Have you ever had vaginal sex (penis in the vagina) or anal sex (penis in the butt) with a woman?

( ) No

( ) Yes

( ) I prefer not to answer

( ) Don't know

Have you ever had oral sex (mouth on the penis) or anal sex (penis in the butt) with a man?

( ) No

( ) Yes

( ) I prefer not to answer

( ) Don't know

Section A: Demographics

What is the highest level of education you completed?

( ) Never attended school

( ) Less than high school

( ) Some high school

( ) High school diploma or GED

( ) Some college, Associate’s Degree, or Technical Degree

( ) College, post graduate or professional school

( ) I prefer not to answer

( ) Don't know

What was your household income last year from all sources before taxes? That is, the total amount of money earned and shared by all people living in your household.

( ) 0 to $19,999 annually (0 to $1667 monthly)

( ) $20,000 to $39,999 annually ($1668 to $3333 monthly)

( ) $40,000 to $74,999 annually ($3334 to $6250 monthly)

( ) $75,000 or more annually ($6251 or more monthly)

( ) I prefer not to answer

( ) Don't know

Including yourself, how many people depend on this income?

____

What kind of health insurance or health care coverage do you currently have?

Health insurance--health plans people get through employment or purchased directly as well as government programs (like Medicare and Medicaid) that provide medical care or help pay medical bills.

Choose all that apply:

[ ] A private health plan (through an employer or purchased directly)

[ ] Medicaid or Medicare

[ ] Some other Medical Assistance program

[ ] TRICARE (CHAMPUS)

[ ] Veterans Administration coverage

[ ] Some other health care plan

[ ] I don't currently have any health insurance

[ ] I prefer not to answer

[ ] Don't know

In the past 12 months, have you seen a doctor, nurse, or other health care provider about your own health?

( ) No

( ) Yes

( ) I prefer not to answer

( ) Don't know

At any of those times you were seen by a doctor or health care provider, were you offered an HIV test? An HIV test checks whether someone has the virus that causes AIDS.

( ) No

( ) Yes

( ) I prefer not to answer

( ) Don't know

Do you consider yourself to be:

( ) Heterosexual or "Straight"

( ) Homosexual or Gay

( ) Bisexual

( ) I prefer not to answer

( ) Don't know

Group B: Stigma & Discrimination

Have you ever told anyone that you are attracted to or have sex with men?

( ) No

( ) Yes

( ) I prefer not to answer

( ) Don't know

Who of the following people have you told that you are attracted to or have sex with men?

	No	Yes	I prefer not to answer	Don't Know
Gay, lesbian, or bisexual friends	( )	( )	( )	( )
Friends who are not gay, lesbian, or bisexual	( )	( )	( )	( )
Family members	( )	( )	( )	( )
Health care provider	( )	( )	( )	( )

During the past 12 months, have any of the following things happened to you because someone knew or assumes you were attracted to men?

	No	Yes	I prefer not to answer	Don't Know
You were called names or insulted	( )	( )	( )	( )
You received poorer services than other people in restaurants, stores, other businesses or agencies	( )	( )	( )	( )
You were treated unfairly at work or school	( )	( )	( )	( )
You were denied or given lower quality health care	( )	( )	( )	( )
You were physically attacked or injured	( )	( )	( )	( )

How strongly do you agree or disagree with the following statement: "Most people in my area are tolerant of gays and bisexuals."

( ) Strongly agree

( ) Agree

( ) Neither agree nor disagree

( ) Disagree

( ) Strongly disagree

( ) I prefer not to answer

( ) Don't know

Section B: Sexual Behavior

Female Sex Partners

The next questions are about having sex with women. For these questions, "having sex" means oral, vaginal, or anal sex. Oral sex means mouth on the vagina or penis; vaginal sex means penis in the vagina; and anal sex means penis in the anus (butt).

In the past 12 months (since [system("date"),format="M"] of 2013) have you had oral, vaginal, or anal sex with a woman?

( ) No

( ) Yes

( ) I prefer not to answer

( ) Don't know

These next questions are about the last time you had oral, vaginal or anal sex with a woman.

Was the woman you had sex with that last time a main partner (someone you felt committed to above anyone else) or a casual partner (someone you didn't feel committed to or don't know very well)?

( ) Main sex partner

( ) Casual sex partner

( ) I prefer not to answer

( ) Don't know

When you had sex that last time, did you have either vaginal or anal sex?

( ) No

( ) Yes

( ) I prefer not to answer

( ) Don't know

The last time you had sex with a woman, did you have either vaginal or anal sex without using a condom?

( ) No

( ) Yes

( ) I prefer not to answer

( ) Don't know

The last time you had sex with this partner, did you know her HIV status?

( ) No

( ) Yes

( ) I prefer not to answer

What was her HIV status?

( ) HIV-negative

( ) HIV-positive

( ) Indeterminate

( ) I prefer not to answer

Male Sex Partners

The next questions are about having sex with men. For these questions, "having sex" means oral or anal sex. Oral sex means he put his mouth on your penis or you put your mouth on his penis. Anal sex means you put your penis in his anus (butt) or he put his penis in your anus (butt).

How old were you the first time you had oral or anal sex with a man?

____

In the past 12 months, since [*system date*] of 2013, with how many different men have you had oral or anal sex?

____

Male Sex Partners (1 Partner)

In the past 12 months, this male partner was a:

( ) Main partner (someone you felt committed to above anyone else)

( ) Casual partner (someone you didn't feel committed to or don't know very well)

( ) I prefer not to answer

( ) Don't know

In the past 12 months, did you have anal sex with this man?

( ) No

( ) Yes

( ) I prefer not to answer

( ) Don't know

In the past 12 months, did you have anal sex without using a condom?

( ) No

( ) Yes

( ) I prefer not to answer

( ) Don't know

Did you know his HIV status?

( ) No

( ) Yes

( ) I prefer not to answer

What was his HIV status?

( ) HIV-negative

( ) HIV-positive

( ) Indeterminate

( ) I prefer not to answer

Male Sex Partners (>1)

In the past 12 months, these male partners were:

( ) Only main partners (you felt committed to above anyone else)

( ) Only casual partners (you didn't feel committed to or don't know very well)

( ) Both main and casual partners

( ) I prefer not to answer

( ) Don't know

In the past 12 months, with any of these male partners did you have anal sex?

( ) No

( ) Yes

( ) I prefer not to answer

( ) Don't know

In the past 12 months, with any of these male partners did you have anal sex without using a condom?

( ) No

( ) Yes

( ) I prefer not to answer

( ) Don't know

In the past 12 months, did you have anal sex without using a condom with a man whose HIV status you did not know?

( ) No

( ) Yes

( ) I prefer not to answer

( ) Don't know

In the past 12 months, did you have anal sex without using a condom with a man who was HIV positive?

( ) No

( ) Yes

( ) I prefer not to answer

( ) Don't know

In the past 12 months, did you have anal sex without using a condom with a man who was HIV negative?

( ) No

( ) Yes

( ) I prefer not to answer

( ) Don't know

Male Sex Partners (Last Sex)

In the next few screens, we're going to ask some questions about your most recent male sex partner--that is, the last guy you had sex with.

To make the questions easier to ask, we'd like you to enter in this partner's initials. If you prefer to leave his initials blank, we will refer to him as "XX".

What are the initials of your last male sex partner?

____

When was the last time you had either oral or anal sex with [*initials*]

Month:

( ) January

( ) February

( ) March

( ) April

( ) May

( ) June

( ) July

( ) August

( ) September

( ) October

( ) November

( ) December

Year: ____

Was [*initials*] a main partner (someone you felt committed to above anyone else) or a casual partner (someone you didn't feel committed to or don't know very well)?

( ) Main sex partner

( ) Casual sex partner

( ) I prefer not to answer

( ) Don't know

That last time you had sex with [*initials*] did you have receptive anal sex where he put his penis in your anus (you were the bottom)?

( ) No

( ) Yes

( ) I prefer not to answer

( ) Don't know

During that last time you had receptive anal sex, did [*initials*] use a condom?

( ) No

( ) Yes

( ) I prefer not to answer

( ) Don't know

Did [*initials*] use the condom the whole time?

( ) No

( ) Yes

( ) Prefer not to answer

( ) Don't know

When you had sex that last time, did you have insertive anal sex where you put your penis in his anus (you were the top)?

( ) No

( ) Yes

( ) I prefer not to answer

( ) Don't know

During insertive anal sex that last time, did you use a condom?

( ) No

( ) Yes

( ) I prefer not to answer

( ) Don't know

Did you use a condom the whole time?

( ) No

( ) Yes

( ) I prefer not to answer

( ) Don't know

Before or during the last time you had sex with [*initials*], did you use:

( ) Alcohol

( ) Drugs

( ) Both alcohol and drugs

( ) Neither one

( ) I prefer not to answer

( ) Don't know

For the next questions, a drink of alcohol is a 12 oz beer, a 5 oz glass of wine, or a 1.5 oz. shot of liquor. A 40 oz beer would count as 3 drinks. A cocktail with 2 shots would count as 2 drinks.

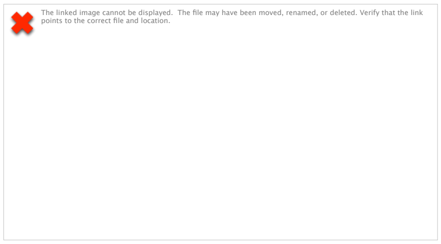

How many alcoholic drinks did you have before or during sex the last time you had sex with [*initials*]?

____

Which drugs did you use the last time you had sex with [*initials*]? Check all that apply.

[ ] Marijuana

[ ] Powdered cocaine

[ ] Poppers

[ ] X or Ecstasy

[ ] Painkillers (Oxycontin, Vicodin, Percocet)

[ ] Downers (Valium, Ativan, Xanax)

[ ] Crystal meth (tina, crank, ice)

[ ] Hallucinogens (LSD, mushrooms)

[ ] Special K (ketamine)

[ ] GHB

[ ] Heroin

[ ] Speedballs (heroin and cocaine together)

[ ] Crack cocaine

[ ] Other drug: ____

[ ] I prefer not to answer

[ ] Don't know

The last time you had sex with [*initials*], did you know his HIV status?

( ) No

( ) Yes

( ) I prefer not to answer

What was [*initials*]'s HIV status?

( ) HIV-negative

( ) HIV-positive

( ) Indeterminate

( ) I prefer not to answer

Group C: Male Sex Partners

When you had sex that last time, did you give [*initials*] things like money or drugs in exchange for sex?

( ) No

( ) Yes

( ) I prefer not to answer

( ) Don't know

When you had sex that last time, did [*initials*] give you things like money or drugs in exchange for sex?

( ) No

( ) Yes

( ) I prefer not to answer

( ) Don't know

Was [*initials*] younger than you, older than you, or the same age as you?

[ ] Younger

[ ] Older

[ ] Same age

[ ] I prefer not to answer

[ ] Don't know

What was [*initials*]'s age?

____

Which of the following best describes [*initials*]'s ethnic background?

[ ] American Indian or Alaska Native

[ ] Asian

[ ] Black or African American

[ ] Hispanic or Latino

[ ] Native Hawaiian or other Pacific Islander

[ ] White

[ ] I prefer not to answer

[ ] Don't know

As far as you know, has [*initials*] ever injected drugs like heroin, cocaine, or speed?

Would you say he:

( ) Definitely did not

( ) Probably did not

( ) Probably did

( ) Definitely did

( ) I prefer not to answer

( ) Don't know

As far as you know, has [*initials*] ever used crystal meth (tina, crank, ice)?

( ) Definitely did not

( ) Probably did not

( ) Probably did

( ) Definitely did

( ) I prefer not to answer

( ) Don't know

Did you have sex with [*initials*] one time ('one night stand'), or more than one time?

( ) One time

( ) More than one time

( ) I prefer not to answer

( ) Don't know

How long have you been having a sexual relationship with [*initials*]?

____

( ) Days

( ) Months

( ) Years

As far as you know, during the time you were having a sexual relationship with [*initials*] , did he have sex with other people?

Would you say he:

( ) Definitely did not

( ) Probably did not

( ) Probably did

( ) Definitely did

( ) I prefer not to answer

( ) Don't know

During the time you were having a sexual relationship with [*initials*], did you have sex with other people?

( ) No

( ) Yes

( ) I prefer not to answer

( ) Don't know

As far as you know, during the past 12 months when you were having a sexual relationship with [*initials*], did he have sex with other people? Would you say he:

( ) Definitely did not

( ) Probably did not

( ) Probably did

( ) Definitely did

( ) I prefer not to answer

( ) Don't know

During the past 12 months when you were having a sexual relationship with [*initials*], did you have sex with other people?

( ) No

( ) Yes

( ) I prefer not to answer

( ) Don't know

Where did you first meet this [*initials*]?

( ) Internet

( ) Chat line

( ) House party

( ) Bar/Club

( ) Circuit party or Rave

( ) Cruising area

( ) Adult bookstore

( ) Place of worship (e.g. church, synagogue, mosque)

( ) Bath house, sex club or sex resort

( ) Private sex party

( ) Somewhere else

( ) I prefer not to answer

( ) Don't know

In the past 12 months, how often have you gone to a place where gay men hangout, meet or socialize? These could include bars, clubs, social organizations, parks, gay businesses, bookstores, sex clubs, etc.

( ) Never

( ) More than once a day

( ) Once a day

( ) More than once a week

( ) Once a week

( ) More than once a month

( ) Once a month

( ) Less than once a month

( ) I prefer not to answer

( ) Don't know

In the past 12 months, how often have you used the internet to meet or socialize with gay men? This includes visiting social network websites (such as Facebook or Myspace), websites directed towards gay men (such as Manhunt or Gay.com), dating websites, or the use of mobile social applications (such as Foursquare or Grindr).

( ) Never

( ) More than once a day

( ) Once a day

( ) More than once a week

( ) Once a week

( ) More than once a month

( ) Once a month

( ) Less than once a month

( ) I prefer not to answer

( ) Don't know

Section C: Substance Use

Alcohol Use

For the next set of questions, a drink of alcohol is a 12 oz beer, a 5 oz glass of wine, or a 1.5 ox shot of liquor. A 40 oz beer would count as 3 drinks. A cocktail with 2 shots would count as 2 drinks.

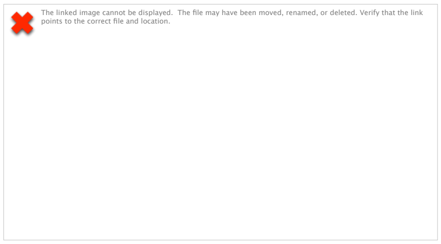

In the past 12 months, how often did you drink any alcohol such as beer, wine, malt liquor, or hard liquor?

( ) Never

( ) More than once a day

( ) Once a day

( ) More than once a week

( ) Once a week

( ) More than once a month

( ) Once a month

( ) Less than once a month

( ) I prefer not to answer

( ) Don't know

In the past 12 months, how often did you have 5 or more alcoholic drinks in one sitting?

( ) Never

( ) More than once a day

( ) Once a day

( ) More than once a week

( ) Once a week

( ) More than once a month

( ) Once a month

( ) Less than once a month

( ) I prefer not to answer

( ) Don't know

Injection Drug Use

The next screens are about injection drug use. This means injecting drugs yourself or having someone who isn't a health care provider inject you.

Have you ever in your life shot up or injected any drugs other than those prescribed for you? By shooting up, we mean anytime you might have used drugs with a needle, either by mainlining, skin popping, or muscling.

( ) No

( ) Yes

( ) I prefer not to answer

( ) Don't know

In the past 12 months, on average, how often did you inject?

( ) Never

( ) More than once a day

( ) Once a day

( ) More than once a week

( ) Once a week

( ) More than once a month

( ) Once a month

( ) Less than once a month

( ) I prefer not to answer

( ) Don't know

Which drug do you inject most often?

( ) Speedball - Heroin and cocaine together

( ) Heroin, by itself

( ) Cocaine, by itself

( ) Crack

( ) Crystal, meth, tina, crank, ice

( ) Something else (Specify)

( ) I prefer not to answer

( ) Don't know

Non-Injection Drug Use

The next questions are about drugs that you may have used but did not inject.

In the past 12 months, have you used any non-injection drugs (drugs you did not inject), other than those prescribed for you.

( ) No

( ) Yes

( ) I prefer not to answer

( ) Don't know

In the past 12 months, which drugs did you use? (Check all that apply.)

[ ] Marijuana

[ ] Powdered cocaine (smoked or snorted)

[ ] Poppers (amyl nitrate)

[ ] X or Ecstasy

[ ] Painkillers (Oxycontin, Vicodin, Percocet)

[ ] Downers (Valium, Ativan, Xanax)

[ ] Crystal meth (tina, crank, ice)

[ ] Hallucinogens (LSD, mushrooms)

[ ] Special K (ketamine)

[ ] GHB

[ ] Crack cocaine

[ ] Other drug: ____

[ ] I prefer not to answer

[ ] Don't know

In the past 12 months, how often did you use [*drug name*]?

( ) More than once a day

( ) Once a day

( ) More than once a week

( ) Once a week

( ) More than once a month

( ) Once a month

( ) Less than once a month

( ) I prefer not to answer

( ) Don't know

In the past 12 months, have you used Viagra, Levitra, or Cialis?

( ) No

( ) Yes

( ) I prefer not to answer

( ) Don't know

Section D: HIV Testing

Have you ever been tested for HIV? An HIV test checks whether someone has the virus that causes AIDS.

( ) No

( ) Yes

( ) I prefer not to answer

( ) Don't know

In the past 2 years, that is since [*system date*] of 2012, how many times have you been tested for HIV?

____

When did you have your most recent HIV test?

Month:

( ) January

( ) February

( ) March

( ) April

( ) May

( ) June

( ) July

( ) August

( ) September

( ) October

( ) November

( ) December

Year:: ____

When you got tested in [*test date*] where did you get tested?

( ) Private doctor's office

( ) HIV counseling and testing site

( ) Public health clinic/community health clinic

( ) Street outreach program/mobile unit

( ) Sexually transmitted disease clinic

( ) Hospital (inpatient)

( ) Correctional facility (jail or prison)

( ) Emergency room

( ) At home

( ) Other

( ) I prefer not to answer

( ) Don't know

HIV Status

What was the result of your most recent HIV test?

( ) Negative

( ) Positive

( ) Never obtained results

( ) Indeterminate

( ) I prefer not to answer

( ) Don't know

Before your test in [*test date*] did you ever test positive for HIV?

( ) No

( ) Yes

( ) I prefer not to answer

( ) Don't know

Was your test in [*test date*] your first positive test?

( ) No

( ) Yes
( ) I prefer not to answer
( ) Don't know

When did you first test positive?

Month:

( ) January
( ) February
( ) March
( ) April
( ) May
( ) June
( ) July
( ) August
( ) September
( ) October
( ) November
( ) December
Year:: ____

Are you currently taking antiretroviral medicines to treat your HIV infection?

( ) No
( ) Yes
( ) I prefer not to answer
( ) Don't know

What is the main reason you are not currently taking any antiretroviral medicines?

( ) Not currently going to a health care provider for my HIV infection

( ) CD4 count and viral load are good

( ) Don't have money or insurance for antiretroviral medicines

( ) Don't want to take antiretroviral medicines

( ) Other

( ) I prefer not to answer

( ) Don't know

Group A: Anti-HIV Meds & Antiretrovirals

Researchers are studying whether antiretroviral medicines could possibly be taken to prevent HIV.

Researchers are studying whether anti-HIV medicine (also called antiretrovirals)--a pill-- could possibly be taken to prevent HIV infection.

Before today, have you ever heard of people who do not have HIV taking antiretroviral medicines or anti-HIV medicines, a pill, to keep from getting HIV?

( ) No

( ) Yes

( ) I prefer not to answer

( ) Don't know

In the past 12 months (since [*system date*] of 2013), have you given your antiretroviral medicines to a sex partner who was HIV-negative because you thought it might keep them from getting HIV?

( ) No

( ) Yes

( ) I prefer not to answer

( ) Don't know

In the past 12 months, have you taken anti-HIV medicines after sex because you thought it would keep you from getting HIV?

( ) No

( ) Yes

( ) I prefer not to answer

( ) Don't know

In the past 12 months, have you taken anti-HIV medicines before sex because you thought it would keep you from getting HIV?

( ) No

( ) Yes

( ) I prefer not to answer

( ) Don't know

Did you get any of the anti-HIV medicines you took from the following people or places?

	**No**	**Yes**	**Prefer not to answer**	**Don't know**
Doctor or other health care provider	( )	( )	( )	( )
Sex partner, friend, relative, or acquaintance	( )	( )	( )	( )
Internet	( )	( )	( )	( )
Some other place	( )	( )	( )	( )

Would you be willing to take anti-HIV medicines every day to lower your chances of getting HIV?

( ) No

( ) Yes

( ) I prefer not to answer

( ) Don't know

nPEP

nPEP, or non-occupational post-exposure prophylaxis, means taking drugs that are normally used to treat HIV/AIDS after you have had a potential exposure to keep your body from taking in the HIV virus. For instance, if you have unprotected sex with someone who is HIV positive, taking these drugs afterwards can significantly reduce your chance of actually becoming infected with the virus. In order to work properly, the drugs must be taken every day for 28 days after exposure and the pills must be started no later than 3 days after the incident – But the sooner the better. There has been shown to be an 80% reduction in transmission. Some people experience nausea, fatigue or weight loss while on the pills.

Have you previously heard about nPEP, taking pills used to treat HIV for 28 days after an exposure in order to prevent HIV infection?

( ) No

( ) Yes

Have you ever been prescribed nPEP?

( ) No

( ) Yes

Have you ever used nPEP?

( ) No

( ) Yes

When did you most recently use nPEP?

Month

( ) January

( ) February

( ) March

( ) April

( ) May

( ) June

( ) July

( ) August

( ) September

( ) October

( ) November

( ) December

Year:: ____

If you had a high risk exposure, such as a condom breaking with an HIV-positive partner, how likely would you be to use nPEP if it were offered at no cost to you?

( ) Very likely

( ) Likely

( ) Neutral

( ) Unlikely

( ) Very unlikely

If you were taking nPEP, how acceptable would it be for you to do a finger-stick blood test at home (like a person with diabetes taking their blood sugar), so your provider could monitor whether or not you are taking the medications correctly?

( ) Very acceptable

( ) Acceptable

( ) Neutral

( ) Unacceptable

( ) Very unacceptable

You indicated you were not likely to consider taking nPEP. What is the main reason for this?

( ) I am not at risk of being infected with HIV

( ) I don't mind if I get HIV

( ) I would not want to go to the trouble of seeking treatment

( ) I don't think nPEP works well at preventing HIV

( ) I want to avoid medication side effects

( ) Other reason, please specify:: ____

Acute Infection

Now we would like to ask you a few questions about your understanding of HIV infection.

Do you think that after a person is infected with HIV, they can show symptoms within a month of being infected?

( ) No

( ) Yes

( ) Don't know

How long after the exposure to infection do you think these symptoms occur?

( ) 1 Day

( ) 3 Days

( ) 1 Week

( ) 2-4 Weeks

( ) Don't know

How long do you think these symptoms last?

( ) 1 Day

( ) 3 Days

( ) 1 Week

( ) 2-4 Weeks

( ) Don't know

About what percent of people who are infected with HIV show symptoms shortly after they are infected?

0 ________________________[__]____________________________ 100

Which of the following do you think are symptoms people have shortly after HIV infection?

[ ] Fever

[ ] Fatigue

[ ] Muscle or joint soreness

[ ] Headache

[ ] Excessive thirst

[ ] Frequent urination

[ ] Weight loss

[ ] Sweaty hands or feet

[ ] Don't know

Have you ever had any disease symptoms and worried that you had recently (in the prior month) become infected with HIV?

( ) No

( ) Yes

For the next question, when we say unprotected sex, we mean that you or your partner did not use a condom at all, did not use it the entire time during sex, or that a condom broke during sex.

Please indicate whether you have been in any of the following situations in the last twelve months (check all that apply):

[ ] I had unprotected receptive anal sex (bottom) with a partner who was HIV-positive.

[ ] I had unprotected receptive anal sex (bottom) with a partner whose HIV status I did not know.

[ ] I had unprotected insertive anal sex (top) with a partner who was HIV-positive.

[ ] I had unprotected insertive anal sex (top) with a partner whose HIV status I did not know.

You indicated you had unprotected receptive anal sex (bottom) with a partner who was HIV-positive. Was this:

( ) A one-time event

( ) Something that happened just a few times

( ) Something that happened regularly

You indicated you had unprotected receptive anal sex (bottom) with a partner whose HIV status you did not know. Was this:

( ) A one-time event

( ) Something that happened just a few times

( ) Something that happened regularly

You indicated you had unprotected insertive anal sex (top) with a partner who was HIV-positive. Was this:

( ) A one-time event

( ) Something that happened just a few times

( ) Something that happened regularly

You indicated you had unprotected insertive anal sex (top) with a partner whose HIV status you did not know. Was this:

( ) A one-time event

( ) Something that happened just a few times

( ) Something that happened regularly

You described having a one-time unprotected sex event. If nPEP were available to you after this event, would you have wanted to take it?

( ) No

( ) Yes

PrEP

PrEP stands for pre-exposure prophylaxis. It involves a healthy person taking a pill used to treat HIV in order to prevent being infected with HIV. The pills have to be taken once a day, every day. Some people who take these pills experience side effects. These may include nausea and weight loss, which usually go away after the first month. In rare cases, taking the pill for long periods may damage the kidneys. The medication is prescribed by a doctor. Taking this medication provides only partial protection against HIV infection. So, a person on the medication should still practice other HIV prevention strategies like using condoms every time. For people who take the pill every day, studies have shown that it provides up to 90% protection against HIV infection.

Would you be interested in taking daily anti-HIV pills to prevent HIV infection (PrEP)?

( ) No

( ) Yes

( ) Don't know

Group B: Stigma & Discrimination

How strongly do you agree or disagree with each statement below?

Most people in my area would discriminate against someone with HIV.

( ) Strongly agree

( ) Agree

( ) Neither agree nor disagree

( ) Disagree

( ) Strongly disagree

( ) I prefer not to answer

( ) Don't know

Most people in my area would support the rights of a person with HIV to live and work wherever they wanted to.

( ) Strongly agree

( ) Agree

( ) Neither agree nor disagree

( ) Disagree

( ) Strongly disagree

( ) I prefer not to answer

( ) Don't know

Most people in my area would not be friends with someone with HIV.

( ) Strongly agree

( ) Agree

( ) Neither agree nor disagree

( ) Disagree

( ) Strongly disagree

( ) I prefer not to answer

( ) Don't know

Most people in my area would think that people who got HIV through sex or drug use have gotten what they deserve.

( ) Strongly agree

( ) Agree

( ) Neither agree nor disagree

( ) Disagree

( ) Strongly disagree

( ) I prefer not to answer

( ) Don't know

Group A: Health Conditions & Services

Has a doctor, nurse or other health care provider ever told you that you had hepatitis?

( ) No

( ) Yes

( ) I prefer not to answer

( ) Don't know

What type or types of hepatitis have you had? Check all that apply.

[ ] Hepatitis A

[ ] Hepatitis B

[ ] Hepatitis C

[ ] Other

[ ] I prefer not to answer

[ ] Don't know

Has a doctor, nurse or other health care provider ever told you that you had genital herpes?

( ) No

( ) Yes

( ) I prefer not to answer

( ) Don't know

Has a doctor, nurse or other health care provider ever told you that you had genital warts?

( ) No

( ) Yes

( ) I prefer not to answer

( ) Don't know

Has a doctor, nurse or other health care provider ever told you that you had human papillomavirus or HPV?

( ) No

( ) Yes

( ) I prefer not to answer

( ) Don't know

In the past 12 months (since [*system date*] or 2013), has a doctor, nurse or other health care provider told you that you had gonorrhea?

( ) No

( ) Yes

( ) I prefer not to answer

( ) Don't know

In the past 12 months, (since [*system date*] or 2013), has a doctor, nurse or other health care provider told you that you had chlamydia?

( ) No

( ) Yes

( ) I prefer not to answer

( ) Don't know

In the past 12 months, (since [*system date*] of 2013), has a doctor, nurse or other health care provider told you that you had syphilis?

( ) No

( ) Yes

( ) I prefer not to answer

( ) Don't know

Even though a doctor, nurse, or other health care provider did not tell you that you had Gonorrhea in the past 12 months, (since [*system date*] of 2013), were you tested for gonorrhea?

( ) No

( ) Yes

( ) I prefer not to answer

( ) Don't know

Even though a doctor, nurse, or other health care provider did not tell you that you had Chlamydia in the past 12 months, since (since [*system date*] of 2013), were you tested for Chlamydia?

( ) No

( ) Yes

( ) I prefer not to answer

( ) Don't know

Even though a doctor, nurse, or other health care provider did not tell you that you had Syphilis in the past 12 months, since (since [question("value"), id="231"] of 2013), were you tested for Syphilis?

( ) No

( ) Yes

( ) I prefer not to answer

( ) Don't know

There are vaccines or shots that can prevent some types of hepatitis. Have you ever had a hepatitis vaccine?

( ) No

( ) Yes

( ) I prefer not to answer

( ) Don't know

What type or types of hepatitis vaccine have you had?

( ) Hepatitis A vaccine

( ) Hepatitis B vaccine

( ) Both Hepatitis A and B vaccine

( ) I prefer not to answer

( ) Don't know

A vaccine to prevent human papillomavirus (HPV) infection is available and is called the HPV shot, cervical cancer vaccine, GARDASIL®, or CERVARIX®. Have you ever received the HPV vaccine?

( ) No

( ) Yes

( ) I prefer not to answer

( ) Don't know

How old were you when you received your first dose of the HPV vaccine?

____

Section F. Assessment of Prevention Activities

In the past 12 months, have you gotten any free condoms, not counting those given to you by a friend, relative, or sex partner?

( ) No

( ) Yes

( ) I prefer not to answer

( ) Don't know

In the past 12 months, have you had a one-on-one conversation with an outreach worker, counselor, or prevention program worker about ways to prevent HIV? Don't count the times where you had a conversation as part of an HIV test.

( ) No

( ) Yes

( ) I prefer not to answer

( ) Don't know

In the past 12 months, have you been a participant in any organized session(s) involving a small group of people to discuss ways to prevent HIV? Don't include discussions you had with a group of friends.

( ) No

( ) Yes

( ) I prefer not to know

( ) Don't know

In the past 12 months, how often have you had one-on-one conversations with a main sexual partner about ways to prevent HIV?

( ) Never

( ) Rarely

( ) Sometimes

( ) Often

( ) Very Often

( ) I prefer not to answer

( ) Don't Know

In the past 12 months, how often have you had one-on-one conversations with a casual sexual partner about ways to prevent HIV?

( ) Never

( ) Rarely

( ) Sometimes

( ) Often

( ) Very Often

( ) I prefer not to answer

( ) Don't know

How sure are you that you could get an HIV test within the next 3-6 months if you wanted to?

( ) Very sure I could not

( ) Somewhat sure I could not

( ) Slightly sure I could

( ) Somewhat sure I could

( ) Very sure I could

( ) I prefer not to answer

( ) Refuse to answer

How likely is it that you will get an HIV test within the next 3-6 months?

( ) Very unlikely

( ) Somewhat unlikely

( ) Slightly likely

( ) Somewhat likely

( ) Very likely

( ) I'd prefer not to answer

( ) Refuse to answer

Most gay men I know get tested for HIV at least every 3-6 months

( ) Strongly agree

( ) Agree

( ) Neither agree nor disagree

( ) Disagree

( ) Strongly disagree

( ) I prefer not to answer

( ) Don't know

As far as you know, did you participate in the "Sex is the Question" Survey in 2012?

( ) Yes, I participated in 2012

( ) No, I did not participate in 2012

( ) I'm not sure

For this national study, we are recruiting a large number of men like you. Can you tell us the name of a new or different social networking website where we could reach other men like you who might like to complete this survey?

____

Survey End

Thank you for taking our survey. Your response is very important to us.
