# Supplementary figures and images for: The Annual American Men's Internet Survey of Behaviors of Men Who Have Sex With Men in the United States: Protocol and Key Indicators Report 2013
Source: JMIR Public Health Surveill. 2015 Apr 17;1(1):e3. doi: 10.2196/publichealth.4314 (PMC4869242; doi:10.2196/publichealth.4314)

## Slide 1
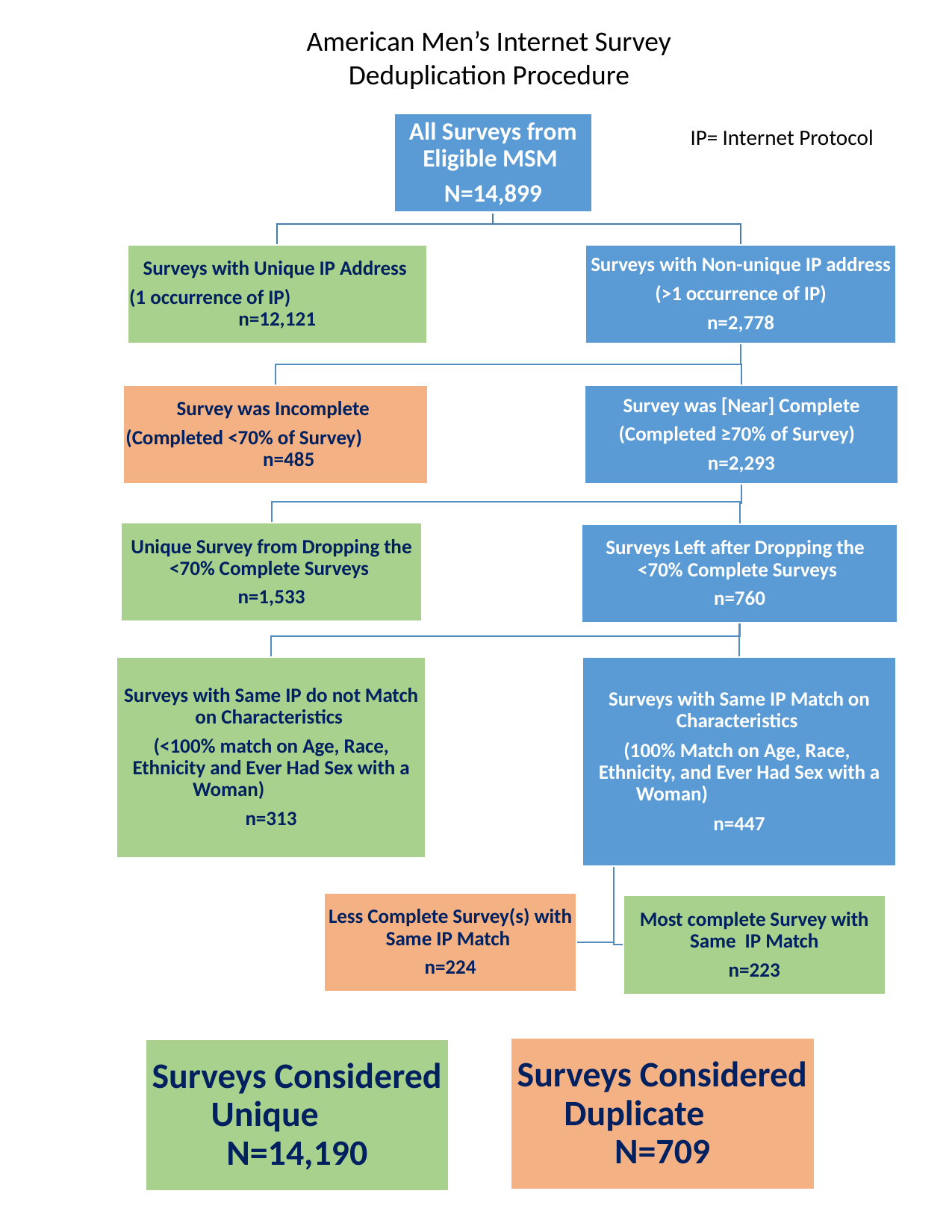

American Men’s Internet Survey
Deduplication Procedure
IP= Internet Protocol

Supplement: Multimedia Appendix 2 [file publichealth_v1i1e3_app2.pptx]
