# Supplementary material for: The Annual American Men's Internet Survey of Behaviors of Men Who Have Sex With Men in the United States: Protocol and Key Indicators Report 2013
Source: JMIR Public Health Surveill. 2015 Apr 17;1(1):e3. doi: 10.2196/publichealth.4314 (PMC4869242; doi:10.2196/publichealth.4314)
Supplement: Multimedia Appendix 3 [file publichealth_v1i1e3_app3.pdf]

Characteristics of MSM Participants in the American Men's Internet Survey by Randomization to Different Question Subsets, United States, 2013

			Randomized Questionnaire						
**Participant Characteristics**	**Core Questionaire**		**Subset A**		**Subset B**		**Subset C**		
	**No.**	**(%)**	**No.**	**(%)**	**No.**	**(%)**	**No.**	**(%)**	**P value[a]**
**Race/Ethnicity**									.999
Black, non-Hispanic	354	(3.41)	118	(3.39)	119	(3.39)	117	(3.45)	
Hispanic	1,084	(10.45)	366	(10.52)	362	(10.33)	356	(10.49)	
White, non-Hispanic	8,076	(77.83)	2,702	(77.69)	2,739	(78.12)	2,635	(77.66)	
Other or multiple races	863	(8.32)	292	(8.40)	286	(8.16)	285	(8.40)	
**Age (years)**									.564
18-24	1,982	(19.10)	673	(19.35)	636	(18.14)	673	(19.83)	
25-29	1,515	(14.60)	504	(14.49)	528	(15.06)	483	(14.24)	
30-39	1,918	(18.48)	624	(17.94)	659	(18.80)	635	(18.72)	
40 or older	4,962	(47.82)	1,677	(48.22)	1,683	(48.00)	1,602	(47.21)	
**Region**									.247
Midwest	2,078	(20.03)	676	(19.44)	750	(21.39)	652	(19.22)	
Northeast	2,050	(19.76)	711	(20.44)	686	(19.57)	653	(19.25)	
South	3,558	(34.29)	1,209	(34.76)	1,169	(33.34)	1,180	(34.78)	
West	2,503	(24.12)	827	(23.78)	836	(23.84)	840	(24.76)	

U.S. dependent areas		8	(0.08)		1	(0.03)		4	(0.11)		3	(0.09)	
**NHBS City Resident**													.400
Yes		3,268	(31.49)		1,071	(30.79)		1,132	(32.29)		1,065	(31.39)	
No		7,109	(68.51)		2,407	(69.21)		2,374	(67.71)		2,328	(68.61)	
**Population Density**													.788
Rural		3,833	(36.94)		1,292	(37.15)		1,279	(36.48)		1,262	(37.19)	
Urban		6,544	(63.06)		2,186	(62.85)		2,227	(63.52)		2,131	(62.81)	
**Self-reported HIV Status**													.656
Positive		1,113	(10.73)		382	(10.98)		384	(10.95)		347	(10.23)	
Negative		7,657	(73.79)		2,544	(73.15)		2,579	(73.56)		2,534	(74.68)	
Unknown		1,607	(15.49)		552	(15.87)		543	(15.49)		512	(15.09)	
**Recruitment Website Type**													.130
Gay social networking		802	(7.73)		294	(8.45)		253	(7.22)		255	(7.52)	
General gay interest		1,958	(18.87)		627	(18.03)		651	(18.57)		680	(20.04)	
General social networking		5,336	(51.42)		1,798	(51.70)		1,838	(52.42)		1,700	(50.10)	
Geospatial social networking		2,281	(21.98)		759	(21.82)		764	(21.79)		758	(22.34)	
**TOTAL**		**10,377**			**3,478**			**3,506**			**3,393**		

MSM = men who have sex with men; NHBS = National HIV Behavioral Surveillance System

[a] Chi-square testing difference in characteristics between randomized questionnaire subsets.
