# Supplementary material for: The Annual American Men's Internet Survey of Behaviors of Men Who Have Sex With Men in the United States: Protocol and Key Indicators Report 2013
Source: JMIR Public Health Surveill. 2015 Apr 17;1(1):e3. doi: 10.2196/publichealth.4314 (PMC4869242; doi:10.2196/publichealth.4314)
Supplement: Multimedia Appendix 4 [file publichealth_v1i1e3_app4.pdf]

American Men's Internet Survey (AMIS):

Online HIV Behavioral Survey of Men Who Have Sex with Men

SAMPLE, 2013

Contents

Introduction
References
Tables

1 Characteristics of participants in an online survey of MSM, SAMPLE, American Men's Internet Survey (AMIS), 2013

2 Sexual behaviors of participants in an online survey of MSM, SAMPLE, American Men's Internet Survey (AMIS), 2013

3 Substance using behaviors of participants in an online survey of MSM, SAMPLE, 2013

4 Sexually Transmitted Infection testing and hepatitis vaccination of participants in an online survey of MSM, SAMPLE, American Men's Internet Survey (AMIS) 2013

5 HIV testing behaviors of HIV-negative or unknown serostatus participants in an online survey of MSM, SAMPLE, American Men's Internet Survey (AMIS), 2013

6 Testing location of last HIV test of HIV-negative or unknown serostatus participants in an online survey of MSM, SAMPLE, American Men's Internet Survey (AMIS), 2013

7 Participation in HIV prevention services of HIV-negative or unknown serostatus participants in an online survey of MSM, SAMPLE, American Men's Internet Survey (AMIS), 2013

8 Knowledge of pre-exposure prophylaxis (PrEP) and willingness to use PrEP of HIV-negative or unknown serostatus participants in an online survey of MSM, SAMPLE, American Men's Internet Survey (AMIS), 2013

Appendix A: Study Questionnaire

Introduction

Men who have sex with men (MSM) contribute to over half of the more than one million adults living with HIV in the United States [1]. Incidence estimates of HIV infection from 2008 to 2011 have shown an increase in the number of new infections among MSM [2], due to factors including high prevalence, sexual risk behaviors, and not being aware of HIV status. The National HIV Behavioral Surveillance System (NHBS), using venue-based sampling to collect risk behavior data from high-risk populations, has found continued sexual risk and drug use behaviors associated with increased HIV risk among MSM [3]. The Centers for Disease Control and Prevention (CDC) recommends annual HIV testing for sexually active MSM (and more frequent for those with multiple sexual partners) [4], but 2011 NHBS data found only about 67% of HIV-negative or unknown status MSM reported an HIV test in the previous 12 months [5]. Monitoring HIV risk and testing behaviors among MSM is important in understanding epidemic trends and the impact of prevention efforts, including behavioral interventions and strategies to increase HIV testing.

An increasing number of MSM are socializing and identifying sexual partners through the internet, and recruiting MSM online offers an opportunity to supplement venue-based studies, such as NHBS, by including at-risk MSM that may not be reached through traditional means, such as bars and clubs [6]. Additionally, online HIV behavioral surveys allow for the collection of data from a large sample of MSM with broad geographic diversity. This report summarizes finding from a large online HIV behavior survey among MSM.

American Men's Internet Survey (AMIS) is a multi-cycle cross-sectional online HIV behavioral survey collecting self-reported data on demographic characteristics, HIV risk behaviors, HIV testing behaviors, and use of HIV prevention services among internet-using MSM in the United States and dependent territories. Participants were recruited online through advertisements (ads), and were consented and screened for eligibility after clicking on the ads. Men were eligible for participation if they were 18 years of age or older, U.S. residents, reported ever having oral or anal sex with a man, and able to complete the survey in English. Men who were eligible to participate in the survey completed a self-administered online survey. The survey consisted of a core questionnaire that every participant answered, 3 different subset questionnaires that participants were randomized to at the start of the survey, and an additional set of questions that were asked of participants from mobile-only social networking ads. The core questions were comprised of the following domains: demographics, sexual behavior, HIV testing history, drug and alcohol use, and HIV prevention services exposure. The randomized question subsets were comprised of the following domains: Subset A - knowledge and use of antiretrovirals for HIV prophylaxis and sexually transmitted disease testing/vaccination; Subset B - disclosure of sexual identity and experiences of stigma; Subset C - additional details about most recent male sex partner. The participants from the mobile-only social networking application received an additional set of questions about a potential mobile HIV prevention application and about acute HIV infection.

The tables in this report include data from the 2013 cycle of AMIS, collected between December 2013 and May 2014, among respondents reporting residence in *SAMPLE*. For demographic categories with small cell sizes, data were collapsed to prevent identification. Study questions and full categories are available in Appendix A. These data were collected as a convenience sample and may not be representative or generalizable to all MSM living in a given state.

References

1. Purcell DW, Johnson CH, Lansky A, Prejean J, Stein R, Denning P*, et al.* Estimating the population size of men who have sex with men in the United States to obtain HIV and syphilis rates. *Open AIDS J* 2012,**6**:98-107.
2. CDC. Estimated HIV incidence in the United States, 2007-2010. In: *HIV Surveillance Supplemental Report*. Atlanta, GA: Centers for Disease Control and Prevention; 2012.
3. Finlayson TJ, Le B, Smith A, Bowles K, Cribbin M, Miles I*, et al.* HIV risk, prevention, and testing behaviors among men who have sex with men--National HIV Behavioral Surveillance System, 21 U.S. cities, United States, 2008. *MMWR Surveill Summ* 2011,**60**:1-34.
4. Branson BM, Handsfield HH, Lampe MA, Janssen RS, Taylor AW, Lyss SB*, et al.* Revised recommendations for HIV testing of adults, adolescents, and pregnant women in health-care settings. *MMWR Recomm Rep* 2006,**55**:1-17; quiz CE11-14.
5. Centers for Disease C, Prevention. HIV testing and risk behaviors among gay, bisexual, and other men who have sex with men - United States. *MMWR Morb Mortal Wkly Rep* 2013,**62**:958-962.
6. Sanchez T, Smith A, Denson D, Dinenno E, Lansky A. Internet-based methods may reach higher-risk men who have sex with men not reached through venue-based sampling. *Open AIDS J* 2012,**6**:83-89.

Table 1. Characteristics of participants in an online survey of MSM, SAMPLE, American Men's Internet Survey (AMIS), 2013

		N	%
Race/ethnicity*	Black, non-Hispanic	36	3.5
	Hispanic	39	3.8
	White, non-Hispanic	889	86.1
	Other or multiple races	69	6.7
Age (years)	18-24	220	21.3
	25-29	147	14.2
	30-39	162	15.7
	40 or older	504	48.8
Education	< HS diploma	17	1.6
	HS diploma or equivalent	117	11.3
	Some college or technical degree	320	31.0
	College degree or postgraduate education	570	55.2
Income	$0-19999	142	13.7
	$20000-39999	224	21.7
	$40000-74999	251	24.3
	$75000 or more	339	32.8
Population density	Rural	434	42.0
	Urban	599	58.0
Self-reported HIV status	Negative	742	71.8
	Positive	97	9.4
	Unknown	194	18.8
Anal sex without a condom with a male partner, past 12 month	No	403	39.0
	Yes	630	61.0
Illicit substances used, past 12 months	No	753	72.9
	Yes	280	27.1
Total		1033	

Percentages may not total 100% due to missing data
MSM=men who have sex with men
HS=high school
****Persons of Hispanic/Latino ethnicity may be of any race***
NOTE: data may be combined or suppressed due to small cell sizes

Table 2. Sexual behaviors with male partners of participants in an online survey of MSM, SAMPLE, American Men's Internet Survey (AMIS), 2013

				Sexual behaviors with male partners in the past 12 months			
				Anal intercourse with a male partner without a condom		Anal intercourse without a condom with last male partner of discordant or unknown HIV status	
			No. in sample	N	%	N	%
**HIV NEGATIVE OR UNKNOWN**	**Age (years)**	18-24	211	136	64.5	26	12.3
		25-29	131	90	68.7	19	14.5
		30-39	155	102	65.8	12	7.7
		40 or older	439	228	51.9	39	8.9
	**Race/ethnicity***	Black, non-Hispanic	28	16	57.1	5	17.9
		Hispanic	32	21	65.6	4	12.5
		White, non-Hispanic	814	478	58.7	80	9.8
		Other or multiple races	62	41	66.1	7	11.3
	**Total**		936	556	59.4	96	10.3
**HIV POSITIVE**	**Age (years)**	18-24	9	7	77.8	5	55.6
		25-29	16	14	87.5	10	62.5
		30-39	7	6	85.7	2	28.6
		40 or older	65	47	72.3	29	44.6
	**Race/ethnicity***	Black, non-Hispanic	8	5	62.5	3	37.5
		Hispanic	7	4	57.1	2	28.6
		White, non-Hispanic	75	60	80.0	38	50.7
		Other or multiple races	7	5	71.4	3	42.9
	**Total**		97	74	76.3	46	47.4

Percentages may not total 100% due to missing data
MSM=men who have sex with men
**Persons of Hispanic/Latino ethnicity may be of any race*
NOTE: data may be combined or suppressed due to small cell sizes

Table 3. Substance using behaviors of participants in an online survey of MSM, SAMPLE, American Men's Internet Survey (AMIS), 2013

				Substance use in the past 12 months			
				Used illicit drugs		Binge drank alcohol	
			No. in sample	N	%	N	%
HIV NEGATIVE OR UNKNOWN	Age (years)	18-24	211	78	37.0	154	73.0
		25-29	131	43	32.8	94	71.8
		30-39	155	40	25.8	106	68.4
		40 or older	439	77	17.5	186	42.4
	Race/ethnicity*	Black, non-Hispanic	28	6	21.4	14	50.0
		Hispanic	32	5	15.6	19	59.4
		White, non-Hispanic	814	206	25.3	476	58.5
		Other or multiple races	62	21	33.9	31	50.0
	Total		936	238	25.4	540	57.7
HIV POSITIVE	Age (years)	18-24	9	5	55.6	7	77.8
		25-29	16	8	50.0	12	75.0
		30-39	7	2	28.6	6	85.7
		40 or older	65	27	41.5	25	38.5
	Race/ethnicity*	Black, non-Hispanic	8	3	37.5	5	62.5
		Hispanic	7	3	42.9	3	42.9
		White, non-Hispanic	75	33	44.0	37	49.3
		Other or multiple races	7	3	42.9	5	71.4
	Total		97	42	43.3	50	51.5

Percentages may not total 100% due to missing data
MSM=men who have sex with men
****Persons of Hispanic/Latino ethnicity may be of any race***
NOTE: data may be combined or suppressed due to small cell sizes

Table 4. Sexually transmitted infection testing and hepatitis vaccination of participants in an online survey of MSM, SAMPLE, American Men's Internet Survey (AMIS), 2013

				Sexually transmitted disease testing in the past 12 months		***Ever received hepatitis vaccination***	
			No. in sample*	***N***	***%***	***N***	***%***
HIV NEGATIVE OR UNKNOWN	***Age (years)***	18-24	68	16	23.5	63	92.6
		25-29	36	16	44.4	36	100.0
		30-39	50	11	22.0	49	98.0
		40 or older	162	35	21.6	143	88.3
	Race/ethnicity*	Black, non-Hispanic	9	4	44.4	8	88.9
		Hispanic	10	4	40.0	10	100.0
		White, non-Hispanic	271	61	22.5	251	92.6
		Other or multiple races	26	9	34.6	22	84.6
	Total		316	78	24.7	291	92.1
HIV POSITIVE	***Age (years)***	18-39	9	7	77.8	9	100.0
		40 or older	21	10	47.6	20	95.2
	Race/ethnicity*	Non-white	10	8	80.0	10	100.0
		White, non-Hispanic	20	9	45.0	19	95.0
	Total		30	17	56.7	29	96.7

Percentages may not total 100% due to missing data
MSM=men who have sex with men
****Persons of Hispanic/Latino ethnicity may be of any race***
*****Subset of full cohort***
NOTE: data may be combined or suppressed due to small cell sizes

Table 5. HIV testing behaviors of HIV-negative or unknown serostatus participants in an online survey of MSM, SAMPLE, American Men's Internet Survey (AMIS), 2013

			**Ever HIV tested**		**HIV tested in the past 12 months**	
		**No. in sample**	**N**	**%**	**N**	**%**
*Race/ethnicity**	Black, non-Hispanic	28	25	89.3	15	53.6
	Hispanic	32	27	84.4	14	43.8
	White, non-Hispanic	814	648	79.6	307	37.7
	Other or multiple races	62	49	79.0	31	50.0
Age (years)	18-24	211	129	61.1	77	36.5
	25-29	131	107	81.7	60	45.8
	30-39	155	139	89.7	71	45.8
	40 or older	439	374	85.2	159	36.2
Education	< HS diploma	15	7	46.7	4	26.7
	HS diploma or equivalent	98	60	61.2	30	30.6
	Some college or technical degree	288	228	79.2	108	37.5
	College degree or postgraduate education	526	446	84.8	222	42.2
Household income	$0-19999	122	96	78.7	47	38.5
	$20000-39999	208	168	80.8	89	42.8
	$40000-74999	227	191	84.1	96	42.3
	$75000 or more	309	255	82.5	118	38.2
Population density	Urban	530	435	82.1	214	40.4
	Rural	406	314	77.3	153	37.7
Health insurance	None	91	73	80.2	31	34.1
	Private only	510	408	80.0	191	37.5
	Public only	60	53	88.3	21	35.0
	Other/Multiple	74	50	67.6	20	27.0
Total		936	749	80.0	367	39.2

Percentages may not total 100% due to missing data
MSM=men who have sex with men
HS=high school
****Persons of Hispanic/Latino ethnicity may be of any race***
NOTE: data may be combined or suppressed due to small cell sizes

Table 6. Testing location of last HIV test of HIV-negative or unknown serostatus participants in an online survey of MSM, SAMPLE, American Men's Internet Survey (AMIS), 2013

	Location of most recent HIV test	
	N	***%***
Private doctor's office	294	39.7
HIV counseling and testing site	118	15.9
Public health clinic/community health clinic	146	19.7
Street outreach program/mobile unit	27	3.6
Sexually transmitted disease clinic	12	1.6
Hospital (inpatient)	27	3.6
Correctional facility (jail or prison)	3	0.4
Emergency room	5	0.7
At home	52	7.0

Percentages may not total 100% due to missing data
MSM=men who have sex with men
NOTE: data may be combined or suppressed due to small cell sizes
NOTE: data may be combined or suppressed due to small cell sizes

Table 7. Participation in HIV prevention services of HIV-negative or unknown serostatus participants in an online survey of MSM, SAMPLE, American Men's Internet Survey (AMIS), 2013

			Prevention services received in the past 12 months					
			Received free condoms		***Participated in group discussion***		***Participated in one-on-one prevention counseling***	
		No. in sample	***N***	***%***	***N***	***%***	***N***	***%***
*Race/ethnicity**	Black, non-Hispanic	28	16	57.1	2	7.1	6	21.4
	Hispanic	32	17	53.1	2	6.3	6	18.8
	White, non-Hispanic	814	322	39.6	35	4.3	100	12.3
	Other or multiple races	62	36	58.1	4	6.5	12	19.4
Age (years)	18-24	211	114	54.0	27	12.8	42	19.9
	25-29	131	68	51.9	6	4.6	21	16.0
	30-39	155	71	45.8	1	0.6	20	12.9
	40 or older	439	138	31.4	9	2.1	41	9.3
Education	< HS diploma	15	5	33.3	0	0.0	3	20.0
	HS diploma or equivalent	98	25	25.5	7	7.1	9	9.2
	Some college or technical degree	288	127	44.1	14	4.9	33	11.5
	College degree or postgraduate education	526	229	43.5	21	4.0	79	15.0
Household income	$0-19999	122	58	47.5	9	7.4	19	15.6
	$20000-39999	208	89	42.8	7	3.4	32	15.4
	$40000-74999	227	100	44.1	7	3.1	31	13.7
	$75000 or more	309	111	35.9	14	4.5	32	10.4
Population density	Urban	530	236	44.5	26	4.9	73	13.8
	Rural	406	155	38.2	17	4.2	51	12.6
Health insurance	None	91	33	36.3	4	4.4	11	12.1
	Private only	510	205	40.2	23	4.5	69	13.5
	Public only	60	18	30.0	3	5.0	7	11.7
	Other/Multiple	74	25	33.8	3	4.1	5	6.8
Total		936	391	41.8	43	4.6	124	13.2

Percentages may not total 100% due to missing data
MSM=men who have sex with men
HS=high school
****Persons of Hispanic/Latino ethnicity may be of any race***
NOTE: data may be combined or suppressed due to small cell sizes

Table 8. Knowledge of PrEP and willingness to use PrEP of HIV-negative or unknown serostatus participants in an online survey of MSM, SAMPLE, American Men's Internet Survey (AMIS), 2013

			Heard of PrEP		Willing to use PrEP	
		No. in sample**	N	%	N	%
*Race/ethnicity**	Black, non-Hispanic	9	4	44.4	4	44.4
	Hispanic	10	5	50.0	5	50.0
	Other or multiple races	26	11	42.3	9	34.6
	White, non-Hispanic	271	95	35.1	119	43.9
Age (years)	18-24	68	21	30.9	31	45.6
	25-29	36	21	58.3	21	58.3
	30-39	50	20	40.0	19	38.0
	40 or older	162	53	32.7	66	40.7
Education	< HS of HS diploma/equivalent	35	5	14.3	16	45.7
	Some college or technical degree	102	29	28.4	44	43.1
	College degree or postgraduate education	175	80	45.7	77	44.0
Household income	$0-19999	38	9	23.7	18	47.4
	$20000-39999	64	15	23.4	23	35.9
	$40000-74999	76	38	50.0	37	48.7
	$75000 or more	113	46	40.7	52	46.0
Population density	Rural	140	44	31.4	59	42.1
	Urban	176	71	40.3	78	44.3
Health insurance	None	30	6	20.0	14	46.7
	Other/Multiple	25	7	28.0	7	28.0
	Private only	175	68	38.9	68	38.9
	Public only	20	5	25.0	9	45.0
Total		316	115	36.4	137	43.4

Percentages may not total 100% due to missing data
MSM=men who have sex with men
HS=high school
PrEP= pre-exposure prophylaxis
****Persons of Hispanic/Latino ethnicity may be of any race***
*****Subset of full cohort***
NOTE: data may be combined or suppressed due to small cell sizes
